# Arginine methylation of human DNA topoisomerase I by PRMT5 facilitates DNA relaxation

**DOI:** 10.1093/nar/gkag503

**Published:** 2026-05-30

**Authors:** Saini Basu, Arpan Bhattacharyya, Muqtada Ali Khan, Uttam Pal, Srijita Paul Chowdhuri, Saumya Ranjan Satrusal, Laura Baranello, Dipak Datta, Benu Brata Das

**Affiliations:** Laboratory of Molecular Biology, School of Biological Sciences, Indian Association for the Cultivation of Science, 2A & B, Raja S. C. Mullick Road, Jadavpur, Kolkata 700032, India; Laboratory of Molecular Biology, School of Biological Sciences, Indian Association for the Cultivation of Science, 2A & B, Raja S. C. Mullick Road, Jadavpur, Kolkata 700032, India; Division of Cancer Biology, Council of Scientific and Industrial Research-Central Drug Research Institute, Lucknow 226031, India; Technical Research Centre, S. N. Bose National Centre for Basic Sciences, Salt Lake, Kolkata 700106, India; Laboratory of Molecular Biology, School of Biological Sciences, Indian Association for the Cultivation of Science, 2A & B, Raja S. C. Mullick Road, Jadavpur, Kolkata 700032, India; Division of Cancer Biology, Council of Scientific and Industrial Research-Central Drug Research Institute, Lucknow 226031, India; Academy of Scientific and Innovative Research, Ghaziabad, Uttar Pradesh 201002, India; Department of Cell and Molecular Biology, Karolinska Institute, Stockholm 17177, Sweden; Division of Cancer Biology, Council of Scientific and Industrial Research-Central Drug Research Institute, Lucknow 226031, India; Academy of Scientific and Innovative Research, Ghaziabad, Uttar Pradesh 201002, India; Laboratory of Molecular Biology, School of Biological Sciences, Indian Association for the Cultivation of Science, 2A & B, Raja S. C. Mullick Road, Jadavpur, Kolkata 700032, India

## Abstract

DNA topoisomerase 1 (Top1) is essential for resolving DNA supercoiling during replication and transcription. Here, we identify protein arginine methyltransferase 5 (PRMT5) as a novel regulator of human Top1 activity *via* symmetric dimethylation at arginine residues R^708^ and R^749^, located in the linker and catalytic domains, respectively. Methylation enhances Top1-mediated strand rotation and DNA relaxation without affecting its DNA binding ability. In contrast, methylation-deficient Top1 mutants (Top1^KK^) display impaired subnuclear mobility and accumulate elevated levels of trapped Top1–DNA covalent complexes (Top1cc) upon camptothecin (CPT) treatment. These defects are independent of PRMT5–Top1 binding but are dependent on PRMT5’s enzymatic activity. Loss of Top1 methylation—*via* point mutation, PRMT5 knockout, or pharmacological inhibition—delays Top1cc resolution and amplifies CPT-induced DNA damage. Strikingly, combining PRMT5 inhibitors (PRMT5i) with Top1 poisons such as irinotecan enhances cytotoxicity across multiple cancer cell types. In a triple-negative breast cancer mouse model, this combination significantly suppresses tumor growth and metastasis, accompanied by increased DNA damage. Our results define PRMT5-driven Top1 arginine methylation as a crucial regulatory mechanism and highlight PRMT5i as a means to potentiate Top1-based cancer treatment.

## Graphical Abstract

**Graphical Abstract:**
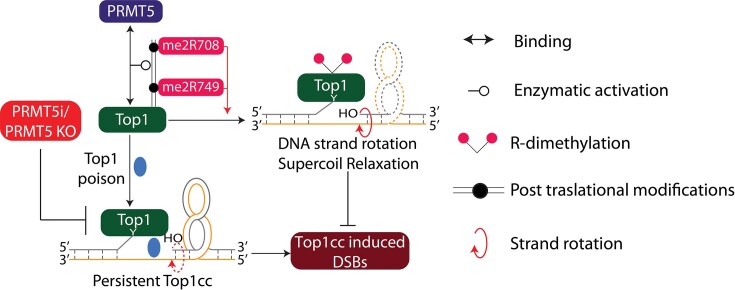

## Introduction

Human DNA topoisomerase 1 (Top1) relieves DNA supercoils generated during replication and transcription. It introduces a transient single-strand break *via* its catalytic tyrosine (Tyr^723^), which attacks the 3′ phosphate of DNA to form a covalent Top1 cleavage complex (Top1cc). DNA relaxation proceeds through controlled strand rotation, where Top1 holds one DNA end while the other rotates, followed by religation to restore DNA integrity [1]. The Top1 religation rate is much faster than the cleavage rate; thus, the covalent enzyme–DNA complexes (Top1cc) are fleeting catalytic intermediates and normally not detectable [2]. In contrast, anticancer drugs such as camptothecin (CPT) and its clinical derivatives [irinotecan (IRI) and topotecan], as well as several non-CPT Top1 inhibitors, trap Top1ccs as irreversible protein–DNA adducts (PDAs). These PDAs are converted into toxic double-strand breaks (DSBs) upon collision with replication or transcription machinery, triggering cell cycle arrest and cell death [3–6].

Top1 is organized into four functional domains [7]: the N-terminal domain (NTD) (1–214 aa) with nuclear localization and protein interaction motifs; the conserved core domain (215–636 aa) forming the cap (subdomains I and II) and lower lobe (subdomain III); the linker domain (637–713 aa), critical for strand rotation and conformational flexibility; and the C-terminal domain (714–765 aa), which harbors the catalytic Y^723^ and residues involved in DNA and drug interactions [8–10].

The linker domain plays a central role in catalysis by acting as a molecular “brake” during strand rotation [11, 12]. It directly contacts DNA and exhibits high structural flexibility, as shown in multiple crystal structures. Point mutations in the linker domain, such as T^729^L, disrupt communication between the linker and C-terminal domain, altering helix interactions and compromising enzyme function. Molecular dynamics (MD) simulations confirm the linker’s importance in interdomain coordination, supercoil relaxation, and CPT sensitivity [11–13].

Top1 function is further regulated by DNA damage-induced post-translational modifications (PTMs) [14]. Phosphorylation by c-Abl (Y^268^) [15] and CK2 (S^506^) [16, 17] and input from PKC and CDK1 modulate Top1 activity and drug response [17]. CPT-induced PARylation by PARP1 promotes Top1 release from stalled replication forks, enhances religation, and shields it from degradation until tyrosyl-DNA phosphodiesterase1 (TDP1) is recruited [18–20]. SUMOylation and PARylation also aid nucleolar clearance of Top1cc [3, 21], while polyubiquitylation enables partial proteasomal degradation, allowing TDP1 to access and resolve trapped Top1ccs [20, 22, 23]. Despite these insights, damage-independent PTMs that regulate Top1’s basal catalytic activity remain poorly understood.

Arginine methylation by protein arginine methyltransferases (PRMTs) is a key regulatory mechanism in DNA repair, cell cycle control, and signal transduction [24–27]. PRMTs transfer methyl groups from S-adenosyl methionine (SAM) to generate asymmetric (Type I), symmetric (Type II), or monomethylated (Type III) arginine residues [26, 27]. PRMT5, the major symmetric dimethyltransferase, modifies both histone (H3, H4) [28] and non-histone proteins, including TDP1, RAD9, FEN1, RuvBL1, 53BP1, and RNA polymerase II [29–32]. It enhances TDP1 catalytic activity and proteostasis, facilitating Top1cc repair [32, 33]. Notably, PRMT5 is overexpressed in various cancers, including leukemia, lymphoma, and solid tumors, making it a compelling target for therapy [29, 34]. Several PRMT5 inhibitors (PRMT5i) such as GSK3326595 are currently in clinical trials and show promise in reducing tumor growth and inducing apoptosis [29, 35].

Despite these advances, the role of arginine methylation in regulating Top1’s catalytic function has remained unexplored. Our study identifies PRMT5-catalyzed symmetric dimethylation at two arginines in Top1, R^708^ in the linker domain and R^749^ in the C-terminal domain. Methylation at R^708^ promotes efficient strand rotation, and loss of methylation at either site impairs DNA supercoil relaxation and sensitizes Top1 to CPT trapping. Strikingly, combining PRMT5i and Top1 inhibitors enhances cancer cell death, suppresses tumor growth, and prevents metastasis in a murine breast cancer model, revealing a novel and promising therapeutic strategy.

## Materials and methods

### Expression constructs and site-directed mutagenesis

The EGFP-tagged human Top1, human FLAG-PRMT5, Flag-tagged N-terminal (1–293 aa; N-term) and C-terminal (294–637 aa; catalytic domain) truncated PRMT5 were described previously [3, 32]. The following point mutations—EGFP-Top1^R708K^, EGFP–Top1^R749K^, EGFP–Top1^R708/749K^ (EGFP-Top1^KK^), and FLAG-PRMT5^R368A^ (FLAG-PRMT5^CD^)—were constructed using the “QuikChange” protocol (Stratagene, La Jolla, CA, USA). EGFP-tagged N-terminal Top1 truncated construct (1–215 aa; N-term) was generated by polymerase chain reaction amplification using full-length EGFP-Top1 and was cloned in the mammalian expression vectors pEGFPN2 (CLONTECH) under the restriction sites of BamH1 and EcoR1. All PCR-generated constructs were further confirmed by DNA sequencing.

### Cell culture, treatment, transfections, and CRISPR/Cas9-mediated gene knockout

Cells were cultured at 37°C under 5% CO_2_ in Dulbecco’s modified Eagle’s medium supplemented with 10% fetal bovine serum (Life Technologies, Rockville, MD, USA). Human embryonic kidney (HEK293), human colon carcinoma (HCT116), and human breast cancer (MCF7) cell lines were obtained from the Developmental Therapeutics Program (NCI, NIH/USA). TDP1^+/+^ and TDP1^−/−^ primary mouse embryonic fibroblast (MEF) cells were a kind gift from Dr Cornelius F Boerkoel (University of British Columbia, Vancouver, British Columbia, Canada). MCF10A was a kind gift from Dr Somsubhra Nath (Presidency University, Kolkata). CRISPR knockout PRMT5 cells (PRMT5^−/−^) were generated in the laboratory by Bhattacharjee *et al*. [36] The CRISPR-based PRMT5 gene knockout was performed in MCF7 cells as described previously [37, 38]. Briefly, the pSpCas9(BB)-2A-GFP (PX458) vector containing the PRMT5 guide RNA (5′ CCTGAATTGCGTCCCCGAAATAG 3′) targeting exon 1 was used to transfect MCF7 using Lipofectamine 2000. GFP-expressing cells were sorted using a FACS cell sorter, and an isogenic clone was generated from a single cell. The loss of PRMT5 protein expression in these clones was confirmed by western blot analysis.

HCT116CMVOsTIR1mAIDTop1 (HCT116Top1_mAID) cells were maintained in media containing 1µg/ml puromycin and 125 µg/ml hygromycin B. For targeted degradation of Top1, HCT116Top1_mAID cells were incubated with 500 µM auxin at 37 °C for 3 h, and to inhibit basal degradation, cells were treated with 100 µM auxinole as a control [39, 40]. Cells were treated with the indicated concentrations of drugs as indicated in the figures. For cycloheximide (CHX) experiments, cells were treated with CHX at a final concentration of 100 µg/ml for the time points indicated in the figure. Plasmid DNAs—EGFP^VC^, EGFP-Top1^WT^, EGFP-Top1^KK^, EGFP-Top1 N-term, FLAG-PRMT^WT^, FLAG-PRMT5 NTD (1–293 aa; N-term), FLAG-PRMT5 catalytic domain (294–637 aa), and FLAG-PRMT5^CD^ (FLAG-PRMT5^R368A^)—were transfected in the cultured cells with TransIT-X2 Dynamic Delivery System (Mirus) according to manufacturer’s instructions. Expression of the proteins was confirmed by western blotting.

### Cell extracts, immunoblotting, and immunoprecipitation

The preparation of whole-cell extracts, immunoprecipitation, and immunoblotting were executed as described previously [32, 33, 41]. Briefly, cells were lysed in a lysis buffer (10 mM Tris–HCl, pH 8, 150 mM NaCl, 0.1% sodium dodecyl sulfate (SDS), 1% NP-40, and 0.5% Na-deoxycholate containing complete protease inhibitor cocktail) (Roche Diagnostics, Indianapolis, IN) and phosphatase inhibitors (Phosphatase Inhibitor Cocktail 1 from Sigma). The lysates were thoroughly mixed and incubated at 4°C for 2 h, followed by centrifugation at 12 000 × *g* at 4°C for 20 min. The resulting supernatants were collected, aliquoted, and stored at −80°C.

For immunoprecipitation, cells were lysed in a lysis buffer (50 mM Tris–HCl, pH 7.4, 300 mM NaCl, 0.4% NP-40, 10 mM MgCl_2_, 0.5 mM dithiothreitol (DTT) supplemented with protease and phosphatase inhibitors). Supernatants of cell lysates were collected after centrifugation at 15 000 × *g* at 4°C for 20 min and precleared with 50μl of protein A/G-PLUS agarose beads (Santa Cruz, CA, USA). About 5 mg of precleared lysates and 50 μl of protein A/G-PLUS agarose beads were incubated overnight at 4°C with indicated antibodies (2–5 μg/ml). The isolated immunocomplexes were recovered by centrifugation, washed thrice with lysis buffer, and subjected to electrophoresis. Gel electrophoresis was carried out on 10% Tris-glycine gels, and immunoblottings were performed following standard procedures. Immunoreactivity was detected using ECL chemiluminescence reaction (Amersham) under ChemiDoc™ MP System (Bio-Rad, USA), and densitometric analyses of immunoblots were performed using ImageJ software.

### Mass spectrometry analysis of PRMT5

Immunoprecipitation of ectopic FLAG–PRMT5 complexes was performed using anti-FLAG antibody. The immunoprecipitated samples were then subjected to tryptic digestion at 37°C overnight. Subsequently, the digested samples were lyophilized, reconstituted, and fractionated using strong cation exchange liquid chromatography. Finally, the fractions obtained were subjected to mass spectrometry (MS) analysis, following previously described methods [32, 41]. The MS proteomics data have been deposited to the ProteomeXchange Consortium *via* the PRIDE repository with dataset identifier PXD057645.

### Proximity ligation assay

Duolink proximity ligation assay (PLA) fluorescence assay (Sigma–Aldrich, Cat# DUO92101) was performed as per the manufacturer’s protocols. In brief, MCF7 cells were seeded onto coverslips and treated with CPT for 3 h. Following treatment, cells were washed with 1× phosphate buffered saline (PBS), fixed with 4% paraformaldehyde in PBS for 15 min at 4°C, and permeabilized for 15 min at 4°C using 0.25% Triton X-100 in PBS. The coverslips were then blocked with Duolink blocking solution and incubated overnight with specified antibodies diluted in Duolink antibody diluents. Subsequently, they were incubated with PLUS and MINUS PLA probes, followed by ligation and amplification. Coverslips were then washed and mounted using a mounting medium containing 4′,6-diamidino-2-phenylindole (DAPI) [20]. Images were acquired using a Leica TCS-SP8 confocal laser-scanning microscope (Germany) with a 63×/1.4 NA oil objective. Images were collected and processed using the Leica software and sized in Adobe Photoshop 7.0.

### Recombinant protein purification

Recombinant His-tagged PRMT5 proteins were expressed using pET28a-PRMT5 expression vectors. BL21 (DE3) *Escherichia coli* cells were transformed with the respective plasmids, and a single colony was inoculated into 20 ml of LB medium containing 50 μg/ml kanamycin and incubated at 37°C for 12 h. The overnight culture was scaled up to 1 l and grown until the OD_600_ reached ∼1.0. Protein expression was induced with 1 mM isopropyl β-D-1-thiogalactopyranoside, followed by overnight incubation at 37°C. Cells were harvested and resuspended in 20 ml lysis buffer (20 mM Tris–HCl, 500 mM NaCl, pH 7.5), vortexed intermittently for 4 h, and further lysed by probe sonication on ice at 30% amplitude with 30 s pulses (10 s on, 20 s off) for 30 min. The lysate was centrifuged at 13 000 rpm for 30 min at 4°C, and the clarified supernatant was subjected to Ni-NTA affinity chromatography to purify the His-tagged proteins. Bound proteins were eluted using elution buffer containing 20 mM Tris–HCl, 500 mM NaCl, and 300 mM imidazole (pH 7.5). For PRMT5, the 6×His-tag was removed by thrombin cleavage according to the manufacturer’s instructions. The purified proteins were concentrated using an Amicon Ultra-15 centrifugal filter unit (10 kDa cutoff, Millipore), aliquoted, and stored at −80°C until further use [36]. The recombinant human His-tagged-Top1 was purified from Sf-9 insect cells infected with the recombinant baculovirus (a kind gift from Dr Yves Pommier, NIH/NCI/USA) as described previously [42].

### Ni^2+^–NTA agarose co-immobilization binding assay

Protein–protein interaction between His-tagged Top1 and untagged PRMT5 was examined using a Ni^2+^–NTA agarose pull-down assay. Briefly, 10 µg of purified hexa-histidine-tagged Top1 was incubated with equimolar PRMT5 in 100 µl of reconstitution buffer containing pre-equilibrated Ni^2+^-NTA agarose beads. The reaction mixture was incubated at 4°C for 2.5 h with gentle rotation to facilitate complex formation. Following incubation, beads were collected by centrifugation and washed twice with 500 µl of reconstitution buffer supplemented with 10 and 20 mM imidazole, respectively, to remove non-specifically bound proteins. Bound protein complexes were eluted with 50 µl of reconstitution buffer containing 250 mM imidazole. Eluted fractions were resolved by 10% SDS–polyacrylamide gel electrophoresis (PAGE) and visualized by Coomassie Brilliant Blue staining [43].

### *In vitro* methylation assays

The *in vitro* methylation assays were performed as previously described [32, 36]. Briefly, PRMT5 was immunoprecipitated and incubated with recombinant His-tagged Top1 proteins (1 μg each) in methylation buffer containing 50 mM Tris–HCl (pH 8.5), 5 mM MgCl_2_, and 4 mM DTT. The reaction mixture was supplemented with 100 μM unlabeled S-(5′-adenosyl)-L-methionine chloride dihydrochloride (SAM; A7007, Sigma) and incubated at 30°C for 2 h. The reactions were terminated by addition of 2× SDS loading buffer (Invitrogen) followed by boiling for 5 min. The methylated products were resolved by SDS–PAGE, transferred onto PVDF membranes, and analyzed by immunoblotting using an anti-symmetrically dimethylated arginine (SDMA) antibody.

### Purification of GFP-Top1 variants by immunoaffinity purification

Immunoprecipitation was performed as described previously [32, 33, 41]. Briefly, HEK293 or auxin-treated HCT116Top1_mAID cells expressing EGFP-Top1 variants (EGFP–Top1^WT^ or EGFP–Top1^KK^) were lysed with lysis buffer. Supernatants of cell lysates were collected after centrifugation at 15 000 × *g* at 4°C for 20 min and precleared with protein A/G-PLUS agarose beads (Santa Cruz, CA, USA). The precleared lysates and protein A/G-PLUS agarose beads were incubated overnight at 4°C with the indicated anti-GFP antibody (2–5 μg/ml). The isolated immunocomplexes were recovered by centrifugation and washed 3–4 times with lysis buffer before elution with 0.1 M glycine (pH 2.9). Eluted proteins were analyzed by immunoblotting [44].

### *In vitro* topoisomerase I DNA relaxation assay

The type I DNA topoisomerases were assayed by decreased mobility of the relaxed isomers of supercoiled pBS(SK+) DNA in agarose gel. The relaxation assays were carried out as described previously [3, 45–47]. Briefly, immunoaffinity-purified EGFP-Top1^WT^ and EGFP-Top1^KK^, whole-cell extracts of PRMT5^+/+^ and PRMT5^−/−^ cells, or PRMT5i-treated or untreated MCF7 cells were used as a source of endogenous Top1 for the time-dependent DNA relaxation experiments. The immune complexes with anti-IgG antibody served as a control. The relaxation assays were performed in the relaxation buffer [25 mM Tris–HCl, pH 7.5, 5% glycerol, 0.5 mM DTT, 10 mM MgCl_2_, 50 mM KCl, 25 mM ethylenediaminetetraacetic acid, and 150 mg/ml bovine serum albumin (BSA)] and supercoiled plasmid pBS (SK+) DNA (85%–95% were negatively supercoiled, with remainder being nicked circles). The amount of supercoiled monomer (SM) DNA band fluorescence after staining with ethidium bromide (EtBr) (0.5 mg/ml) was quantified by using the Bio-Rad ChemiDoc^TM^ MP system under UV illumination (Bio-Rad Quantity One software).

### Electrophoretic mobility shift assay

Analysis of Top1–DNA interaction by electrophoretic mobility shift assay (EMSA) was carried out as described previously [43]. Briefly, a 5′-Cy5-labeled 25-mer oligonucleotide 1 (5′- GAAAAAAGACTTAGAAAAATTTTTA-3′) containing a Top1 binding motif was annealed with oligonucleotide 2 (5′-TAAAAATTTTTCTAAGTCTTTTTTC-3′). The DNA binding assay was performed in a 25 µl reaction volume containing 1 nM of the annealed/duplex oligonucleotide in 100 mM Tris–Cl, pH 8.5, 10 mM ethylenediaminetetraacetic acid (EDTA), and 1 M KCl. Increasing concentrations of immunoaffinity-purified EGFP-Top1^WT^ and EGFP-Top1^KK^, ranging from 250 ng/µl to 2 µg/µl, were incubated with the duplex at 4°C for 1 h. Following incubation, samples were resolved on an 8% non-denaturing polyacrylamide gel in 1× Tris-Boric acid-EDTA buffer (TBE) buffer at 4°C to assess DNA–protein complex [48, 49].

### Computational methods

The structure of the human topoisomerase 1 (UniProt: TOP1_HUMAN; range 201–765) bound with 22-base-pair DNA (5′-AAAAAGACTTGGAAAAATTTTT-3′) was modeled with AlphaFold 3.0, followed by covalent modifications and arginine (R^708^ and R^749^) methylation using Schrodinger Maestro (Academic Release 2020–4). The AlphaFold-generated model was used as the available crystal structures in the Protein Data Bank contained either a mutation, missing residues in the loop region connecting the linker domain, or intercalated ligands in the DNA. The generated model was compared with the existing crystal structure, and the protein Cα standard deviation (SD) was <2 Å. Protonation states and the hydrogen bonding networks of amino acid residues were optimized for pH 7.4 using the PROPKA method during the protein preparation stage as implemented in Schrodinger Maestro [50]. Both the methylated and unmethylated complexes were placed in a cubic simulation box of 120 Å edge length. There were at least 10 Å buffer regions on each side of the protein, and the periodic images of the protein were at least 20 Å apart from each other. The box was filled with a pre-optimized simple point charge water model [51]. The complex was charge-neutralized by adding 23 Na^+^ ions. The simulations were run in an optimized potential for liquid simulations force field [52, 53]. The systems were first equilibrated through a series of short simulations with restraint on the solute, as implemented in the Desmond routine and described in detail earlier [54]. The final simulation was run for 100 ns as an NPT ensemble with a constant number of atoms, isotropic standard atmospheric pressure, and a defined temperature of 300 K (27°C; room temperature). A position restraint of 1 kcal/mol/Å^2^ was kept on the scissile strand of the DNA during the production run. The atomic positions were saved at 100 ps intervals, and a total of 1000 such snapshots were saved. The simulation trajectory was then analyzed to compare the interactions of the methylated and unmethylated Top1 with DNA and the fluctuations in the protein backbone due to methylation. To predict the collective motions of the protein, normal modes were calculated using the NMWizard plugin in VMD (Visual Molecular Dynamics, version 1.9.4a51), which interfaces with ProDy (version 1.10.10). The Anisotropic Network Model (ANM) method was applied to calculate the dominant motions [55]. The full MD trajectories of the protein in both methylated and unmethylated states were used. Before the normal mode calculations, protein backbone atoms (C, N, and Cα) were extracted from the MD trajectories and aligned to the first frame to remove global translational and rotational motions. The elastic network model was constructed considering default ProDy parameters with a cutoff distance of 15 Å, and uniform spring constants were applied between all residue pairs within the cutoff. For each system, the first 10 non-trivial modes were computed. The mobility plots were generated from the first non-trivial mode (Mode 1), which captured the dominant collective motion for the system. No normalization or scaling was applied to the resulting fluctuations. Per-residue mobilities reflect the predicted amplitude of motion for each Cα atom along the first mode. The analysis was repeated for both methylated and unmethylated systems to compare the differential flexibility and mobility patterns associated with methylation.

### Immunocytochemistry and confocal microscopy

Immunofluorescence staining and confocal microscopy were conducted as described previously [33, 41, 56, 57]. In brief, cells were cultured on chamber slides (Thermo Scientific™ Nunc™ Lab-Tek™ II Chamber slides) and treated with drugs, followed by fixation with 4% paraformaldehyde for 10 min at room temperature and permeabilized with 0.25% Triton X-100 for 15 min at 4°C. Primary antibodies against GFP, γH2AX, or Top1cc were detected using anti-rabbit or anti-mouse IgG secondary antibodies labeled with Alexa 488/568 (Invitrogen). Primary antibodies were used at a dilution of 1:300, and the secondary antibodies at a 1:500 dilution. DAPI (Vector Laboratories, Burlingame, CA, USA) was used to mount the cells and was examined under a Leica TCS-SP8 confocal laser-scanning microscope (Germany) with a 63×/1.4 NA oil objective. Images were captured and processed with Leica software and resized using Adobe Photoshop 7.0. The fluorescence intensity per nucleus for the different proteins of interest was quantified with ImageJ Software by measuring the fluorescence intensities normalized to the number of cells counted.

### Immuno complex of enzyme bioassay

Detection of trapped EGFP-Top1 variants and nuclear Top1-cleavage complexes was carried out by immuno complex of enzyme (ICE) bioassay as described previously [58]. Briefly, EGFP^−/−/VC^, EGFP-Top1^−/−/WT^, and EGFP–Top1^−/−/KK^, or PRMT5^+/+^ and PRMT5^−/−^ cells (5 × 10^6^), or MCF7 cells were either untreated or treated with the indicated drug concentration and were lysed by DNAzol reagent (Invitrogen, USA). The lysates were mixed with 0.4 ml of 100% ethanol, incubated for 5 min at −20°C, and cleared by centrifugation (12 000 × *g* for 10 min). The pellet was dissolved in 8 mM NaOH (freshly made), and varying concentrations of DNA were spotted onto a nitrocellulose membrane (Millipore, USA) using a slot-blot vacuum system (Bio-Rad, USA). Immunoblotting was performed using anti-Top1cc-specific antibodies. Anti-double-stranded DNA (dsDNA) was used for loading control. Immunoblots were visualized using enhanced chemiluminescence reactions on a ChemiDoc^TM^ MP System.

### Photobleaching experiments

Photobleaching experiments were performed as described previously [3, 58] using a confocal laser scanning microscope (Leica TCS-SP8) with a 63×/1.4 NA oil objective, and the microscope was equipped with a CO_2_-controlled on-stage heated environmental chamber set to 37°C. Fluorescence recovery after photobleaching (FRAP) analyses were performed with auxin-treated HCT116Top1_mAID cells expressing EGFP-Top1 variants (EGFP–Top1^WT^ or EGFP–Top1^KK^) on chamber cover glass (Genetix, India) and treated with or without drugs as indicated. For FRAP analysis, a subnuclear region was bleached using a 488 nm laser for 30 ms and photographed at intervals of 500 ms thereafter. Successive images were captured for 60 s after bleaching, demonstrating the level of recovery of fluorescence in the bleached areas. Relative fluorescence intensities of the bleached region were corrected for background signals. To generate the FRAP curves, the fluorescence signal measured in a region of interest (ROI) was individually normalized to the pre-bleach signal in the ROI according to the following equation: ROI = (*I*_t_ − *I*_bg_)/ (*I*_o_ − *I*_bg_) × 100, where *I*_o_ is the intensity in the ROI during pre-bleach, *I*_t_ is the intensity in the ROI at time point t, and *I*_bg_ is the background signal determined in a region outside of the cell nucleus.

### FRAP kinetic modeling and fitting

FRAP data fitted with the two-state binding model given by Sprague *et al*. (equation 1) [59].


\begin{eqnarray*}
\mathrm{frap}\left( t \right) & \approx & \left( {{{F}_{\mathrm{ eq}}} + {{C}_{1\mathrm{ eq}}}} \right)\left[ {{{e}^{\frac{{ - {{\tau }_{1\mathrm{ eff}}}}}{{2t}}}}\left( {{{I}_0}\left( {\frac{{{{\tau }_{1\mathrm{ eff}}}}}{{2t}}} \right) + {{I}_1}\left( {\frac{{{{\tau }_{1\mathrm{ eff}}}}}{{2t}}} \right)} \right)} \right]\\&& + {{C}_{2\mathrm{ eq}}}\left( {1 - {{e}^{ - {{k}_{2\mathrm{ off}}}t}}} \right).
\end{eqnarray*}

Where frap (*t)*, is the fluorescence intensity at time *t. F*_eq_, *C*_1eq_, *C*_2eq_ are the equilibrium fractions of free and the two binding states, respectively. τ_1eff_ is the effective recovery time; *k*_2off_ is the dissociation rate of the weakly bound state. *I*_0_ and *I*_1_ are the Bessel functions.

Before fitting, the FRAP data were normalized as:

*F*_norm_(*t*) = (*F*(*t*) − *F*_0_)/ (*F*_prebleach_ − *F*_0_)

where *F*_norm_(*t*) is the normalized fluorescence at time *t; F*(*t*) is the observed fluorescence at time *t; F*_prebleach_ is the fluorescence intensity before bleaching and *F*_0_ is the initial post-bleach fluorescence.

### Cleavage assay

Cleavage assay was carried out as described previously [45, 46, 60]. In brief, immunoaffinity-purified complexes EGFP-Top1^WT^ or EGFP-Top1^KK^ from Top1^−/−^ cells were used as source of Top1 and pBS (SK+) supercoiled DNA were incubated in standard reaction mixture containing 50 mM Tris–HCl, pH 7.5, 100 mM KCl, 10 mM MgCl_2_, 0.5 mM DTT, 0.5 mM EDTA, and 30 µg/ml BSA in the presence of various concentrations of CPT at 37°C for 30 min. The reactions were stopped by adding 1% SDS and 150 µg/ml proteinase K and further incubated for 1 h at 37°C. DNA samples were subjected to electrophoresis in 1% agarose gel containing 0.5 g/ml EtBr to resolve more slowly migrating nicked product (Form II) from the supercoiled molecules (Form I).

### Alkaline comet assays

To detect the level of DNA damage in Top1^−/−/WT^ and Top1^−/−/KK^, cells were analyzed by alkaline comet assays in accordance to the manufacturer’s instructions (Trevigen, Gaithersburg, MD) as described previously [32, 33]. Briefly, after drug treatment and removal for the indicated time points, cells were retrieved, mixed with low-melting agarose, and spread onto a prewarmed slide. The slides were then immersed in alkaline lysis buffer at 4°C for 1 h, rinsed with deionized water, and then incubated in an electrophoresis buffer (50 mM NaOH, 1 mM EDTA, and 1% dimethyl sulfoxide) at 4°C for 1 h. Electrophoresis was performed at a constant voltage of 25 V for 30 min at 4°C. Following electrophoresis, the slides were neutralized in neutralization buffer (0.4 M Tris–HCl, pH 7.5), dehydrated in ice-cold 70% ethanol for 5 min, and air-dried. DNA was stained with propidium iodide (Sigma). Comet tail lengths were measured using OpenComet Software for at least 50 cells.

### Cell survival assays

Auxin-treated HCT116Top1_mAID, MCF7, MCF10A, or HCT116 cells were seeded in 96-well plates (BD Biosciences, USA). HCT116Top1_mAID cells were transfected with EGFP^VC^, EGFP-Top1^WT^, or EGFP-Top1^KK^. Following transfection, the HCT116Top1_mAID cells were treated with CPT at the indicated concentrations for 48 h. MCF7, MCF10A, or HCT116 cells were pretreated with fixed concentrations of PRMT5i for 24 h, followed by IRI, PRMT5i, or IRI alone at the specified concentrations for 72 h. Cell survival was assessed using the 3-(4,5-dimethylthiazol-2-yl)-2,5-diphenyl tetrazolium bromide (MTT) (Sigma), as described previously [32, 33, 41]. Plates were analyzed on a Molecular Devices SpectraMax M2 microplate reader at 570 nm. The percent inhibition of cell viability at each drug concentration was calculated with respect to the control. Data represent mean values ± SD for three independent experiments.

### Synergy measurements

MCF7 cells were seeded in 96-well plates in triplicate and treated with IRI or PRMT5i at the indicated concentrations for 72 h. Cell viability was assessed using the MTT assay (Sigma-Aldrich). To evaluate potential synergistic effects, a matrix of dilution series combining the two drugs was prepared, and combinatorial responses were analyzed using the online SynergyFinder Plus tool (https://synergyfinder.org/#!/cite) based on the Loewe reference model [61]. Synergy scores >10 were interpreted as evidence of synergistic interactions between the two drugs.

### Wound healing assay

For the wound healing assay, 500 000 MCF7 cells were seeded into six-well plates and incubated overnight to allow the formation of a confluent monolayer. A straight-line scratch in the cell monolayer has been made using a scratcher [62]. The cells were washed with PBS and were treated with only IRI or PRMT5i or both IRI pre-treated with PRMT5i for the indicated time points. Reference points were randomly selected within a single well at each time interval, and the percentage of the wound area healed was quantified using ImageJ Wound Healing Plugin software.

### Colony formation assay

Auxin-treated HCT116TOP1_mAID or PRMT5^−/−^ cells were seeded at 200 cells per well in six-well plates and were transfected with EGFP^VC^, EGFP-Top1^WT^, or EGFP-Top1^KK^ with TransIT-X2 Dynamic Delivery System (Mirus) according to the manufacturer’s instructions. The transfected cells were treated with CPT for indicated concentration and were washed with PBS after 3 h. Similarly, 4T1 cells were seeded at 200 cells per well in 24-well plates and were treated either with IRI or PRMT5i or IRI + PRMT5i as indicated in the Figure. The cells were incubated for 2 weeks at 37°C, and the media was aspirated, followed by a wash with PBS. The washed cells were fixed with ice-cold methanol for 10 min and stained with 0.5% crystal violet dye for 1 h [62]. The excess stain was rinsed with water, and the plates were left to air dry. The stained colonies were counted by ImageJ Software.

### Animal studies

All animal studies were conducted by following standard principles and procedures approved by the Institutional Animal Ethics Committee (IAEC) of CSIR-Central Drug Research Institute (Protocol Number: IAEC/2024/17). Experimental mice were maintained in IVC cages under pathogen-free conditions, maintained on a 12-h light/12-h dark cycle at a temperature of 24 ± 2°C and a humidity level of 45 ± 5%. The mice were provided with an irradiated standard mouse diet at the CSIR-CDRI Central Laboratory Animal Facility. All studies were conducted using 6-week-old female Balb/c nude mice [62, 63]. For orthotopic inoculation, 4T1 cells tagged with Td-Tomato and Luc (1 × 10^6^ cells in 100 μl) were injected into the mammary fat pad of 4–6-week-old nude Crl: CD1-Foxn1^nu^ female mice. Once tumors reached an average volume of ∼100 mm^3^, mice were randomized into four groups by a blinded independent investigator (*n* = 3 per group): vehicle control (VC), IRI alone, PRMT5i alone, and the combination of IRI and PRMT5i. Tumor volumes at the start of the treatment were comparable across groups. IRI (5 mg/kg) was administered intraperitoneally every other day, while PRMT5i (20 mg/kg) was administered orally daily for 21 days. The combination group received both drugs at the respective doses for the entire experiment. Throughout the study, the tumor was measured with an electronic digital caliper at a regular interval, and the tumor volume was calculated using the standard formula *V* = (*a*^2^ × *b*)/2, where ‘*a*’ is the short and ‘*b*’ is the long tumor axis. At the end of the experiment, mice were sacrificed, and subcutaneous tumors were dissected. Live animal bioluminescent imaging (IVIS spectrum, Perkin Elmer) was performed at the end of the experiment. For *in vivo* imaging studies, tumor-bearing mice were injected intraperitoneally with 150 mg/kg D-Luciferin (10 mg/ ml in PBS). Subsequently, mice were anesthetized with isoflurane, and images were captured with dorsal and ventral positions using the PerkinElmer IVIS system coupled with bioluminescence image acquisition and analysis software. ROI from displayed images were identified on the tumor and metastatic sites and quantified as counts per second (c/s) using Living Image software.

### Immunohistochemical staining and analysis

Formalin-fixed, paraffin-embedded tissue sections were deparaffinized in xylene and rehydrated through a graded ethanol series following standard procedures. Antigen retrieval was performed by boiling the slides in 10 mM sodium citrate buffer (pH 6.0) for 10 min. Slides were then washed three times in distilled water (5 min each), followed by a wash in PBS containing 0.1% Tween-20 for 5 min. Tissue sections were blocked in PBS with 5% BSA for 1h at room temperature and then incubated overnight at 4°C with primary antibodies against Ki-67, γH2AX, or Top1cc. After primary antibody incubation, slides were washed three times in PBS with 0.1% Tween-20 (5 min each) and incubated for 1 h at room temperature with anti-rabbit or anti-mouse IgG secondary antibodies labeled with Alexa 488/568 (Invitrogen). Following secondary antibody incubation, slides were again washed three times in PBS with 0.1% Tween-20 for 5 min each. Primary antibodies were used at a dilution of 1:300, and the secondary antibodies at a 1:500 dilution. DAPI was used to mount the cells, and they were examined under a Leica TCS-SP8 confocal laser-scanning microscope (Germany) with a 63×/1.4 NA oil objective. Images were captured and processed with Leica software and resized using Adobe Photoshop 7.0. The fluorescence intensity per nucleus for the different proteins of interest was quantified with ImageJ Software by measuring the fluorescence intensities normalized to the number of cells counted [64].

### Statistics and reproducibility

All experiments were conducted independently at least three times. Data are expressed as mean ± SD or standard error of the mean (SEM). The number of experiments and the quantified cells per field are detailed in the figure legends. Each experiment (*n*) was performed using different cell variants. Statistical significance (*P* values) was determined using, one-way analysis of variance (ANOVA), two-way ANOVA, and *t*-test, as indicated in the figure legends. Data analysis and graphing were performed using Prism 8 (GraphPad Software) and OriginLab. No specific method was applied to pre-determine sample size, and no data were excluded from the analysis. Images were randomly selected, and data analysis was carried out in a blinded manner.

## Results

### Top1–PRMT5 interaction is independent of DNA damage

**Figure 1.**
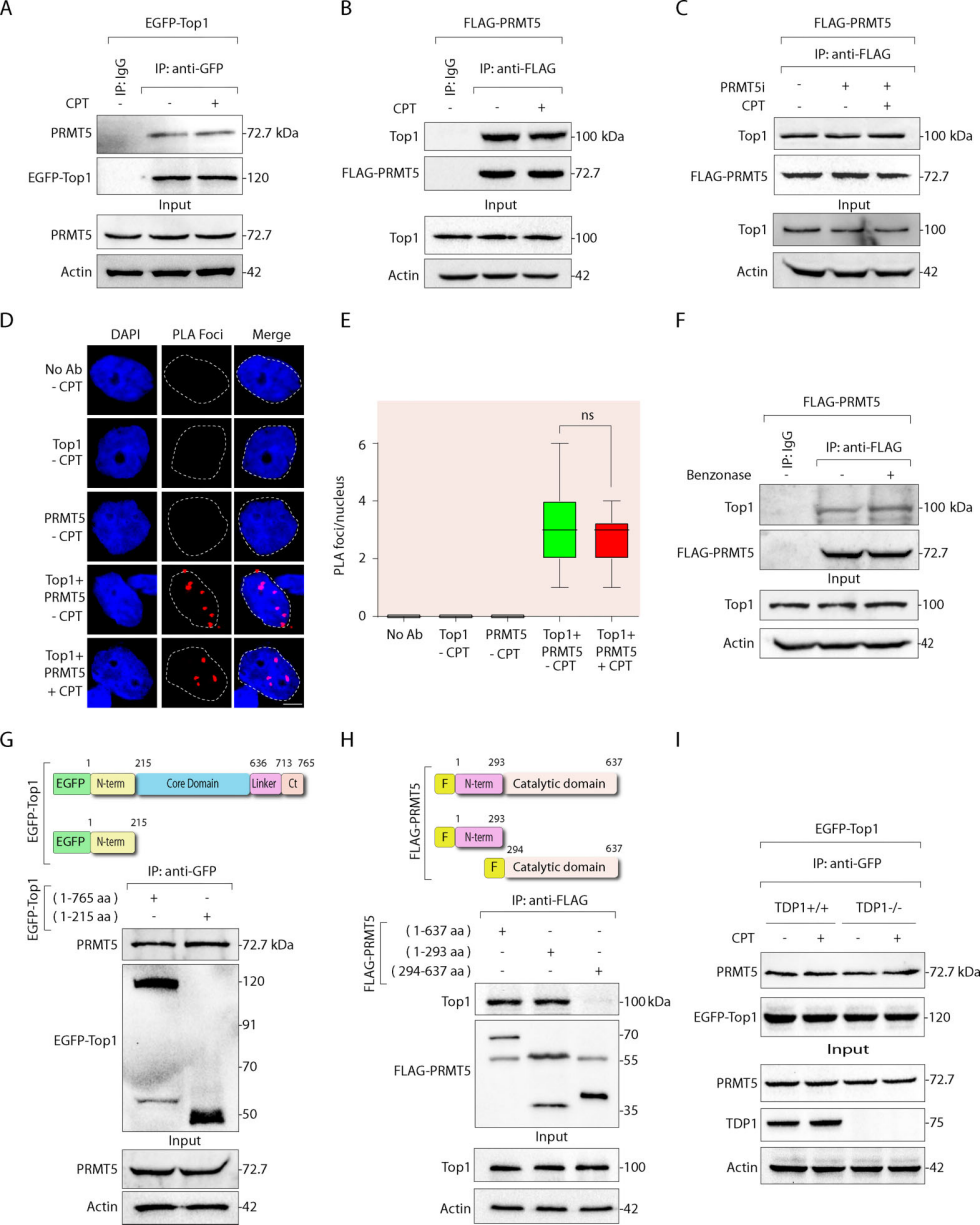
Top1 binds with PRMT5. (**A**) HEK293 cells ectopically expressing EGFP-Top1 were treated with or without CPT (5 µM, 3 h). Following treatment, immunoprecipitation was performed using anti-GFP antibody. The immune complexes were blotted with anti-PRMT5 antibody. Subsequently, the blot was stripped and reprobed using an anti-GFP antibody to indicate uniform loading. Aliquots (10%) of the input show the level of PRMT5 before immunoprecipitation. (**B**) HEK293 cells ectopically expressing FLAG-PRMT5 were treated with or without CPT (5 µM, 3 h) and were immunoprecipitated using anti-FLAG antibody. The immune complexes were blotted with anti-Top1 antibody. The same blot was stripped and reprobed with anti-FLAG antibody to show equal loading. Aliquots (10%) of the input show the level of Top1 before immunoprecipitation. (**C**) Same as panel (B) except the cell lysates were pretreated with or without PRMT5i (5 μM, 24 h) or PRMT5i + CPT (5 μM, 3 h) before co-IP as indicated. (**D**) PLA confirmed the DNA damage-independent binding of endogenous Top1 and PRMT5. MCF7 cells treated with or without CPT (5 µM, 3 h) were fixed for PLA using rabbit PRMT5 antibody, mouse Top1 antibody, or both. The detected PLA foci are represented by red dots. The nuclei were counterstained with DAPI (blue). Scale bar, 5 µm. (**E**) Quantification for the number of PLA foci per nucleus was calculated for 50 nuclei by box-whisker plot. *n* = 3 biological replicates. ns: non-significant (*P* > .05) (*t*-test). (**F**) Same as panel (B), except the cell lysates were pretreated with or without benzonase before co-IP as indicated. (**G**) Schematic representation of EGFP-Top1 constructs illustrating full length (1–765 aa) and truncated NTD (1–215 aa; N-term) of human Top1. EGFP- Top1 variants ectopically expressing in HEK293 cells were immunoprecipitated using anti-GFP antibody. The immune complexes were blotted with anti-PRMT5 antibody, and the same blot was stripped and probed with anti-GFP antibody to show the expression of the EGFP-Top1 variants. Aliquots (10%) of the input reveal the level of PRMT5 prior to immunoprecipitation. (**H**) Schematic representation of Flag-tagged constructs illustrating full-length (1–637 aa), truncated NTD (1–293 aa; N-term), and truncated catalytic domain (294–637 aa) of human PRMT5. FLAG-tagged PRMT5 variants ectopically expressing in HEK293 cells were immunoprecipitated using anti-FLAG antibody. The immune complexes were probed with anti-Top1 antibody. The same blot was stripped and reprobed with anti-FLAG antibody to show the expression of the FLAG-PRMT5 variants. Aliquots (10%) of the input reveal the level of Top1 before immunoprecipitation. (**I**) TDP1^+/+^ and TDP1^−/−^ MEF cells ectopically expressing EGFP-Top1 were treated with or without CPT (5 µM, 3 h) and were immunoprecipitated using anti-GFP antibody. The immune complexes were probed with anti-PRMT5 antibody. The same blot was stripped and reprobed with anti-GFP antibody to show equal loading. Aliquots (10%) of the input demonstrate the level of TDP1 knockout and PRMT5 before immunoprecipitation. Actin served as a loading control.

An unbiased MS analysis of FLAG-PRMT5 pull-down samples identified Top1 as a PRMT5-interacting partner both in the presence and absence of CPT (Supplementary Fig. S1A). To validate this interaction, we immunoprecipitated ectopic EGFP-Top1 from HEK293 cells treated with CPT or left untreated and detected endogenous PRMT5 in the complex (Fig. 1A). Notably, the interaction between EGFP-Top1 and PRMT5 remained unaffected by CPT treatment, suggesting it is independent of CPT-induced Top1cc trapping (Fig. 1A). Next, we performed reverse co-immunoprecipitation (co-IP) experiments in HEK293 cells ectopically expressing FLAG-PRMT5. Endogenous Top1 was detected in the FLAG–PRMT5 complex in both untreated and CPT-treated cells (Fig. 1B). Consistent with Fig. 1A, the interaction between FLAG-PRMT5 and endogenous Top1 was unaltered by CPT treatment (Fig. 1B). We further validated the Top1–PRMT5 interaction in cells by immunoprecipitating endogenous Top1 from HEK293 cells, which co-immunoprecipitated endogenous PRMT5 (Supplementary Fig. S1B). This interaction was detected both in the presence and absence of CPT, indicating that Top1–PRMT5 binding is independent of DNA damage. Under the same conditions, PRMT9, another type II methyltransferase, was not detected in the EGFP-Top1 immunocomplex, confirming the specificity of the Top1–PRMT5 interaction (Supplementary Fig. S1C). Furthermore, the PRMT5i (GSK3326595) did not disrupt the PRMT5–Top1 interaction, indicating it does not require PRMT5 catalytic activity (Fig. 1C).

To validate the *in situ* proximity of Top1 and PRMT5 under endogenous conditions, a PLA was performed in MCF7 cells using anti-Top1 and anti-PRMT5 antibodies. Discrete PLA signals indicative of close proximity between the two proteins, were observed in both untreated and CPT-treated cells, with no signal in negative controls (Fig. 1D), and quantification showed no significant change in PLA foci per nucleus upon CPT treatment (Fig. 1E), supporting co-IP findings (Fig. 1A and B).

To test whether this interaction depends on nucleic acids, co-IPs were performed in the presence of benzonase, which degrades both DNA and RNA [32, 41]. FLAG-PRMT5 continued to co-precipitate Top1 after benzonase treatment (Fig. 1F), confirming that the interaction is nucleic acid-independent. To further confirm direct binding between PRMT5 and Top1, we performed an *in vitro* Ni^2+^-NTA co-immobilization assay using recombinant untagged PRMT5 and His-tagged Top1. PRMT5 was specifically pulled down by His-Top1, supporting a direct physical interaction between the two proteins (Supplementary Fig. S1D).

Given that Top1’s NTD mediates interactions with various nuclear proteins, including TATA-binding protein, nucleolin, SV40 large T antigen, and RNA Pol II [9, 65], we tested whether it binds PRMT5. Co-IP of EGFP-tagged Top1 NTD (1–215 aa) confirmed its ability to pull down endogenous PRMT5 (Fig. 1G). To map the corresponding interaction region on PRMT5, truncated constructs encompassing its FLAG-tagged N-terminal (1–293 aa) and C-terminal (294–637 aa) domains were used. Co-IP showed that the N-terminal region of PRMT5 mediates the interaction with endogenous Top1 (Fig. 1H), suggesting that both proteins interact *via* their N-termini.

Finally, since TDP1 is known to interact with both Top1 and PRMT5 [32, 56, 66], we asked whether TDP1 mediates this interaction. Co-IP of ectopic EGFP-Top1 in TDP1^−/−^ MEFs showed that PRMT5 still co-precipitated with Top1 (Fig. 1I), confirming that the Top1–PRMT5 interaction is independent of TDP1. Together, these results reveal a novel interaction between Top1 and PRMT5, mediated by their respective NTDs, which is independent of TDP1 or CPT-induced Top1cc trapping.

### PRMT5 catalyzes symmetric dimethylation of Top1 at R^708^ and R^749^

**Figure 2.**
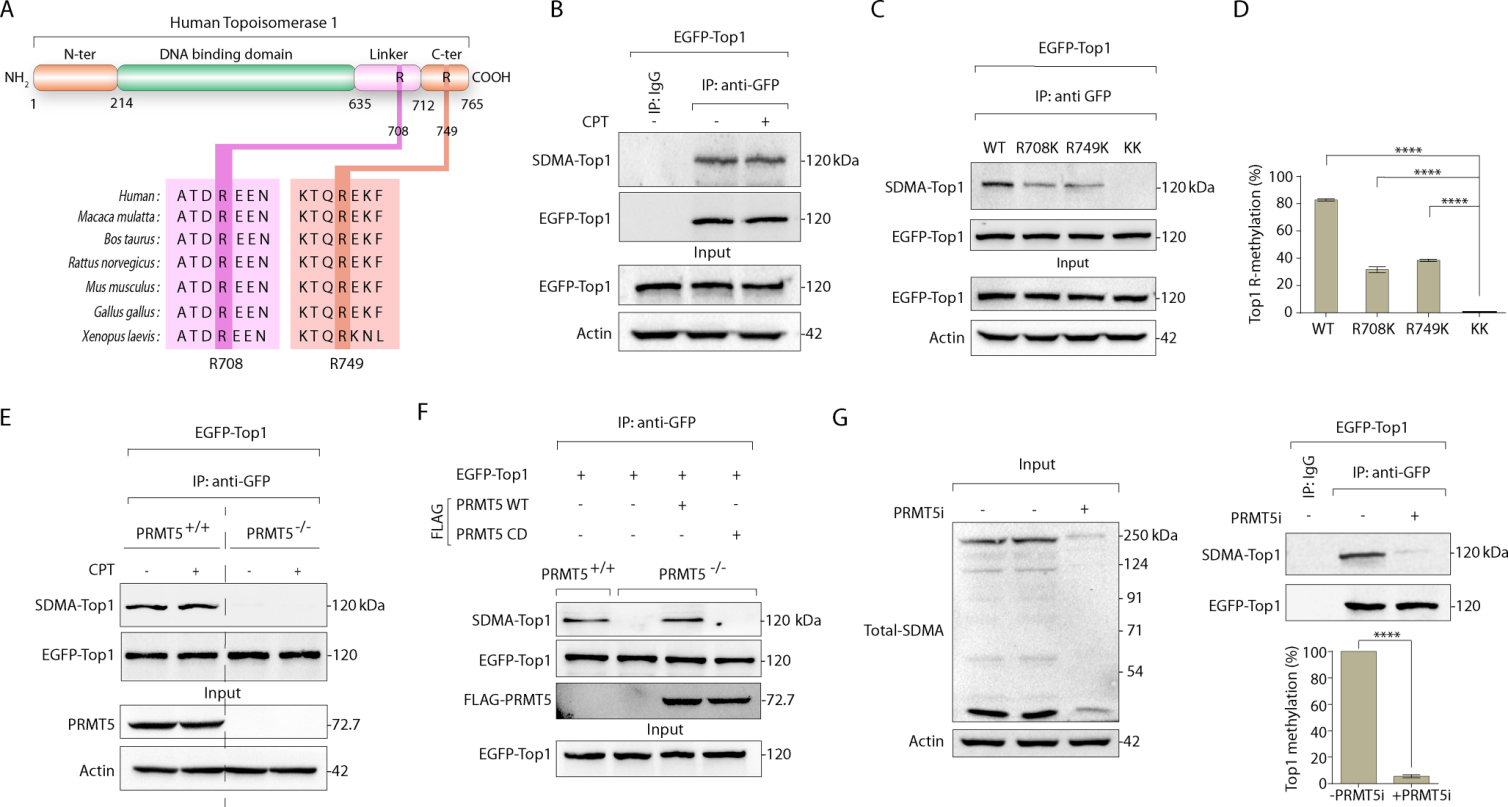
PRMT5 catalyzes the symmetric dimethylation of Top1 at R^708^ and R^749^. (**A**) Schematic representation illustrating the arginine dimethylation sites (R^708^ and R^749^) on human Top1. The phylogenetic conservation of Top1 sequences across human (*Homo sapiens*), monkey (*Macaca mulatta*), cattle (*Bos taurus*), rat (*Rattus norvegicus*), mouse (*Mus musculus*), jungle fowl (*Gallus gallus*), and African clawed frog (*Xenopus laevis*) is evidenced by the alignment of sequences spanning R^708^ and R^749^, as indicated by pink and brown boxes. (**B**) HEK293 cells ectopically expressing EGFP-Top1 were treated with or without CPT (5 µM, 3 h). Immunoprecipitation was performed using an anti-GFP antibody. The immune complexes were blotted with anti-SDMA-specific antibody. The same blot was stripped and reprobed using anti-GFP antibody to indicate equal loading. Control immunoprecipitation using anti-IgG confirms the specificity of the reactions. Aliquots (10%) of the input show the level of EGFP-Top1 before immunoprecipitation. (**C**) To detect arginine methylation of Top1 at R^708^ and R^749^, EGFP-tagged Top1 constructs—EGFP-Top1^WT^, single mutants: EGFP-Top1^R708K^ and EGFP-Top1^R749K^, and double mutant: EGFP-Top1^R708K/R749K^ (EGFP-Top1^KK^)—ectopically expressing in HEK293 cells were immunoprecipitated using anti-GFP antibody. The immune complexes were blotted with anti-SDMA antibody, and the same blot was stripped and reprobed with anti-GFP antibody to show the equal loading. Aliquots (10%) of the input show the level of EGFP-Top1 before immunoprecipitation. (**D**) Densitometry analysis showing symmetric arginine dimethylation level of wild-type and mutant Top1. Top1 arginine methylation was quantified, normalized to EGFP-Top1, and represented as fold change. Error bars represent mean ± SEM, *n* = 3 biological replicates, ****P ≤ .0001 (one-way ANOVA). (**E**) PRMT5 catalyzes the symmetric dimethylation of Top1. PRMT5^+/+^ and PRMT5^−/−^ MCF7 cells ectopically expressing EGFP-Top1 were treated with or without CPT (5 µM, 3 h). Immunoprecipitation was performed using anti-GFP antibody, and the immune complexes were blotted with anti-SDMA antibody. Subsequently, the blot was stripped and reprobed using anti-GFP antibody to indicate uniform loading. Aliquots (10%) of the input show the level of PRMT5 knockout. (**F**) PRMT5^+/+^ and PRMT5^−/−^ MCF7 cells were co-transfected with EGFP-Top1 and FLAG-PRMT5 variants (WT or catalytic dead, CD) as indicated. Immunoprecipitation was performed using an anti-GFP antibody. The immune complexes were blotted with anti-SDMA antibody, and the same blot was stripped and probed with anti-GFP antibody to show the equal loading. To check the expression of FLAG-PRMT5, the blot was probed with anti-FLAG antibody. Aliquots (10%) of the input show the level of EGFP-Top1 before immunoprecipitation. (**G**) HEK293 cells ectopically expressing EGFP-Top1 were treated with or without PRMT5i (5 µM, 24 h) and immunoprecipitated with anti-GFP antibody. The immune complexes were blotted with anti-SDMA-specific antibody. The same blot was stripped and reprobed with anti-GFP antibody to indicate equal loading. Aliquots (10%) of the input were probed with anti-SDMA antibody to check the whole cell SDMA level after PRMT5i treatment. Top1 arginine methylation was quantified and normalized to EGFP-Top1 and represented as fold change. Actin served as a loading control. Error bars represent mean ± SEM (*n* = 3), ****P ≤ .0001 (one-way ANOVA). Asterisks denote statistically significant differences.

MS analysis of endogenous Top1 co-immunoprecipitated with FLAG-PRMT5 identified R^708^ and R^749^ as candidate symmetric dimethylation sites (Supplementary Fig. S2A and B). R^708^ lies within the linker domain, while R^749^ is proximal to the catalytic tyrosine Y^723^. Both residues are evolutionarily conserved from *Xenopus* to humans, suggesting functional relevance across species (Fig. 2A).

To confirm the Top1 arginine methylation, we performed co-IP of ectopic EGFP-Top1 in the presence and absence of CPT and probed with a specific antibody that recognizes SDMA mark on the target protein [32]. Figure 2B shows the SDMA band of Top1, matching the molecular weight of EGFP-Top1, with similar SDMA intensity in both CPT-treated and untreated conditions (Fig. 2B and quantification in Supplementary Fig. S2C). The presence of SDMA signals on EGFP-Top1 in the absence of CPT indicates that Top1 is methylated, independent of DNA damage, further supporting the DNA damage-independent nature of the Top1–PRMT5 interaction (Fig. 1A and B).

We next generated methylation-deficient mutant EGFP-Top1 variants (WT; single mutants: R^708^K, R^749^K; double mutant: R^708^K/R^749^K, or KK), which were ectopically expressed in HEK293 cells and co-immunoprecipitated using an anti-GFP antibody (Fig. 2C). The single mutants, EGFP-Top1^R708K^ and EGFP-Top1^R749K^, showed a significant reduction in SDMA signals, which was completely abolished in the double mutant EGFP-Top1^KK^ (Fig. 2C and quantification in Fig. 2D), confirming symmetric dimethylation at R^708^ and R^749^. We next assessed the role of arginine methylation on Top1 stability or subcellular localization. To examine the role of Top1 arginine dimethylation in Top1 stability, we measured the half-life of the EGFP-Top1^WT^ and EGFP-Top1^KK^ in endogenous Top1-depleted HCT116Top1_mAID (Top1^−/−^) cells complemented either with EGFP-Top1^WT^ (Top1^−/−/WT^), or arginine methylation mutant EGFP-Top1^KK^ (Top1^−/−/KK^) in the presence of protein synthesis inhibitor CHX. Supplementary Fig. S2D revealed no significant difference in protein half-life between the two proteins, indicating that loss of arginine methylation does not alter Top1 stability (Supplementary Fig. S2D and quantification in Supplementary Fig. S2E). Next, we analyzed the subcellular distribution of ectopic EGFP-Top1^WT^ and EGFP-Top1^KK^ by live-cell confocal microscopy in Top1^−/−^ cells. Supplementary Fig. S2F shows that both the proteins localized predominantly in the nucleus, with no detectable differences. The association between EGFP-Top1^KK^ and PRMT5 was unchanged in co-IP experiments (Supplementary Fig. S2G), indicating that the loss of methylation did not impair the Top1–PRMT5 association, Top1-stability or subcellular localization.

To confirm PRMT5 as the methyltransferase, EGFP-Top1 was expressed in PRMT5-knockout (PRMT5^−/−^) MCF7 cells. SDMA signals were lost in PRMT5^−/−^ cells regardless of CPT treatment, and restored by complementing PRMT5^−/−^ cells with FLAG-tagged human PRMT5^WT^, but not by the catalytically dead PRMT5^R368A^ (FLAG-PRMT5^CD^) mutant [67, 68] (Fig. 2E and F). Moreover, pharmacological inhibition of PRMT5 by GSK3326595 in EGFP-Top1-expressing HEK293 cells abolished Top1 methylation (Fig. 2G) under a condition where it abrogates cellular SDMA in cells. To further substantiate Top1 methylation by PRMT5, we performed *in vitro* methylation assays with recombinant His-tagged Top1 as substrates for immunoprecipitated PRMT5 in the presence of SAM. We detected PRMT5-mediated methylation of recombinant Top1 in the presence of SAM (Supplementary Fig. S2H), consistent with Top1 methylation observed in cells (Fig. 2B). Collectively, these results establish that PRMT5 not only interacts but also symmetrically dimethylates Top1 at R^708^ and R^749^.

### Arginine methylation enhances Top1 catalytic activity

**Figure 3.**
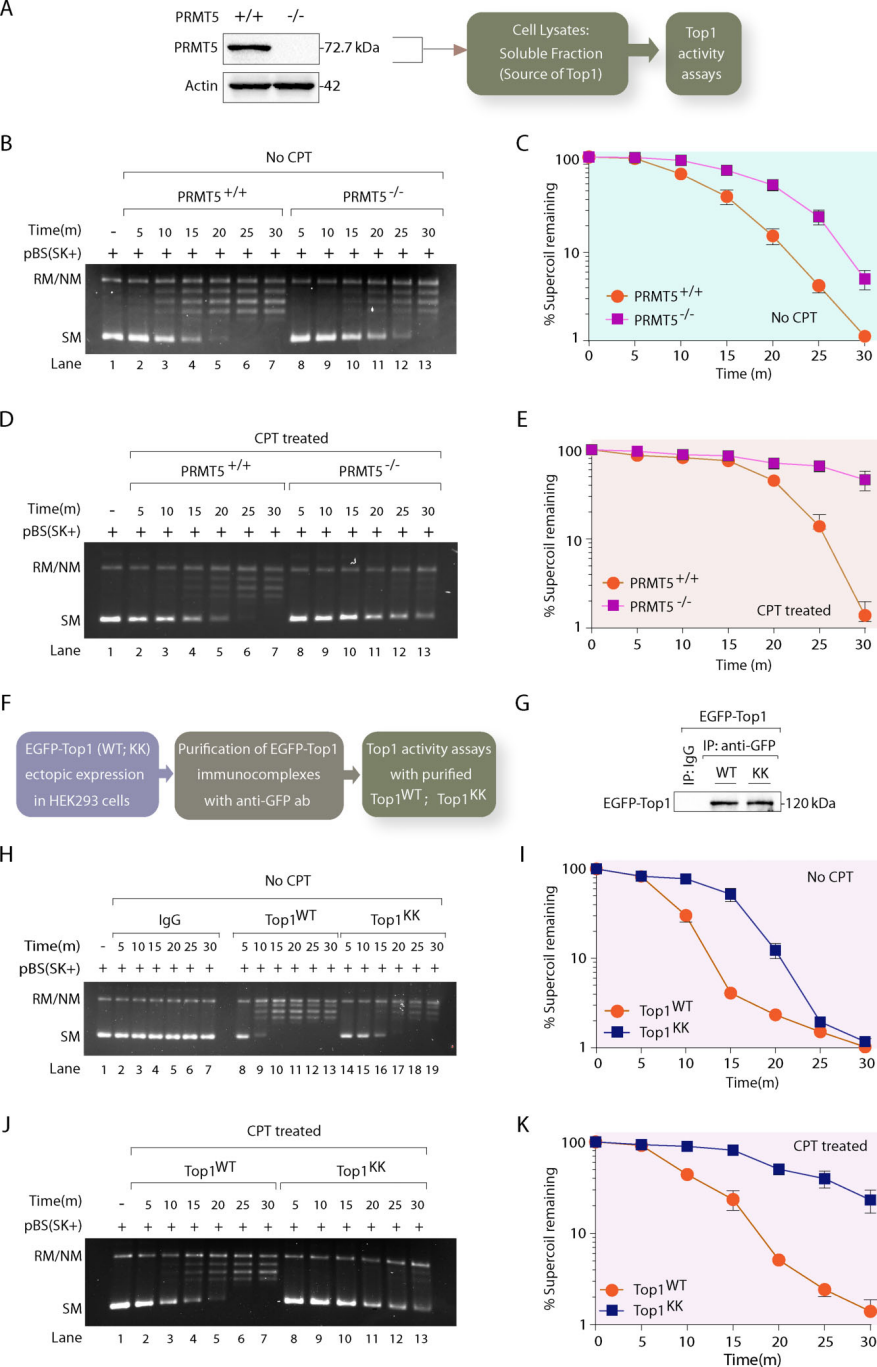
Arginine methylation promotes Top1-mediated DNA relaxation activity. (**A**) Representative western blot showing the level of PRMT5 in PRMT5 ^+/+^ and PRMT5 ^−/−^ MCF7 cells. Actin served as a loading control. Schematic representation showing the preparation of MCF7 whole-cell lysates to be used as the source of endogenous Top1 for Top1 plasmid DNA relaxation activity assays. (**B**) Time-dependent plasmid DNA relaxation activity assay was performed with PRMT5 ^+/+^ and PRMT5 ^−/−^ cell lysates (each reaction volume contains 0.1 μg protein) in the absence of CPT: Lane 1 pBS (SK+) DNA (300 ng); lanes 2–7, equivalent to lane 1 but DNA was incubated with PRMT5 ^+/+^ cell lysates for indicated time points; lanes 8–13, also equivalent to lane 1 but DNA was incubated with PRMT5^−/−^ cell lysates for indicated time points. (**C**) Graphical representation showing the percentage of supercoiled plasmid DNA remaining over time. (**D**) Same as panel (B) except the DNA relaxation activity assay was performed in a time-dependent manner with the PRMT5 ^+/+^ and PRMT5 ^−/−^ cell lysates in the presence of CPT. (**E**) Graphical representation illustrating the percentage of supercoiled plasmid DNA remaining with time. (**F**) Schematic illustration portraying the scheme of immunoaffinity purification of EGFP-Top1^WT^ and EGFP-Top1^KK^ from HEK293 cells for time-dependent Top1 activity assays. (**G**) EGFP-tagged Top1^WT^ and Top1^KK^ constructs ectopically expressing in HEK293 cells were subjected to immunoprecipitation using anti-GFP antibody. The resulting immune complexes were eluted and blotted with anti-GFP antibody. Control immunoprecipitation was conducted with an anti-IgG antibody to confirm the specificity of the reactions. (**H**) Plasmid DNA relaxation activity assay was carried out in time-dependent manner with control IgG, EGFP-Top1^WT^, and EGFP-Top1^KK^ (each reaction volume contains 0.1 μg protein) in the absence of CPT: Lane 1 pBS (SK+) DNA (300 ng); lanes 2–7, equivalent to lane 1 but DNA was incubated with IgG; lanes 8–13, also equivalent to lane 1 but DNA was incubated with EGFP-Top1^WT^, lanes 14–19, identical to lane 1 but DNA was incubated with EGFP-Top1^KK^ for indicated time points. (**I**) Graph illustrating the percentage of supercoiled DNA remaining over time. (**J**) Same as panel (H) except the time-dependent plasmid DNA relaxation activity assay was performed in the presence of CPT. (**K**) Graph representing the percentage of supercoiled DNA remaining over time. All reactions were stopped by adding SDS at a final concentration of 0.5% (w/v) and subsequently subjected to electrophoresis on a 1% agarose gel. Positions of SM, relaxed and nicked monomer (RM/NM) are denoted in the gel. All the experiments were performed three times and expressed as the mean ± SD.

Top1 activity, defined by DNA strand incision, controlled rotation, and religation, can be directly measured *via* plasmid DNA relaxation assays under multiple turnover conditions [1, 69]. To examine the role of Top1 arginine methylation, we used cellular lysates as a source of Top1 from PRMT5^+/+^ and PRMT5^−/−^ MCF7 cells (Fig. 3A), preserving the enzyme in its native state with PTMs and interaction with cofactors [45, 60, 70].

Plasmid DNA relaxation assays [3, 45, 46] revealed a ∼2-fold reduction in Top1 activity from PRMT5^−/−^ lysates compared to PRMT5^+/+^ cells (Fig. 3B and quantification in Fig. 3C), even in the absence of CPT, indicating that arginine methylation intrinsically supports catalytic efficiency. The addition of CPT further impaired relaxation by PRMT5^−/−^ Top1, as evidenced by a ∼3-fold reduction in Top1 activity (Fig. 3D and quantification in Fig. 3E). These results reveal that Top1’s intrinsic arginine methylation enhances its catalytic activity.

To directly validate these findings, we immunoaffinity purified wild-type (EGFP-Top1^WT^) and a methylation-deficient Top1 mutant (EGFP-Top1^KK^) from HEK293 cells (Fig. 3F and G). In the absence of CPT, EGFP-Top1^KK^ showed a similar ∼2-fold delay in plasmid relaxation compared to EGFP-Top1^WT^ (Fig. 3H and quantification in Fig. 3I). This delay was exacerbated by CPT treatment (Fig. 3J and quantification in Fig. 3K), further supporting that that arginine methylation at R^708^ and R^749^ intrinsically promotes Top1-mediated DNA relaxation activity.

Pharmacological inhibition of PRMT5 using GSK3326595 [29, 35] also abrogate Top1 methylation (Supplementary Fig. S3A–D) and demonstrate a ∼2-fold reduction in Top1-mediated DNA relaxation activity, mimicking the phenotypes observed in PRMT5^−/−^ cells (Fig. 3B and C) and EGFP-Top1^KK^ mutants (Fig. 3H and I). Accordingly, CPT treatment further delayed the relaxation of Top1 extracted from cells treated with the PRMT5i (Supplementary Fig. S3E and quantification in Supplementary Fig. S3F). Notably, the GSK3326595 did not directly affect Top1 plasmid DNA relaxation activity when added during the assay (Supplementary Fig. S3G and quantification in Supplementary Fig. S3H), excluding off-target effects. Taken together, these findings suggest that Top1 methylation at R^708^ and R^749^ residues enhance plasmid DNA relaxation activity.

### Top1 arginine methylation facilitates efficient DNA relaxation by promoting strand rotation

The delayed plasmid relaxation activity observed in the arginine methylation-deficient Top1 mutant (Top1^KK^) (Fig. 3H and J) suggests potential defects either in DNA binding or strand rotation. To test these possibilities, we compared the non-covalent DNA-binding affinities of EGFP-Top1^WT^ and EGFP-Top1^KK^ using a Cy5-labeled 25-mer duplex oligonucleotide in EMSA (Supplementary Fig. S4A) [43, 48, 49]. Both variants exhibited comparable DNA binding, indicating that the delayed relaxation is likely due to impaired strand rotation rather than altered DNA-binding affinity (Supplementary Fig. S4B).

**Figure 4.**
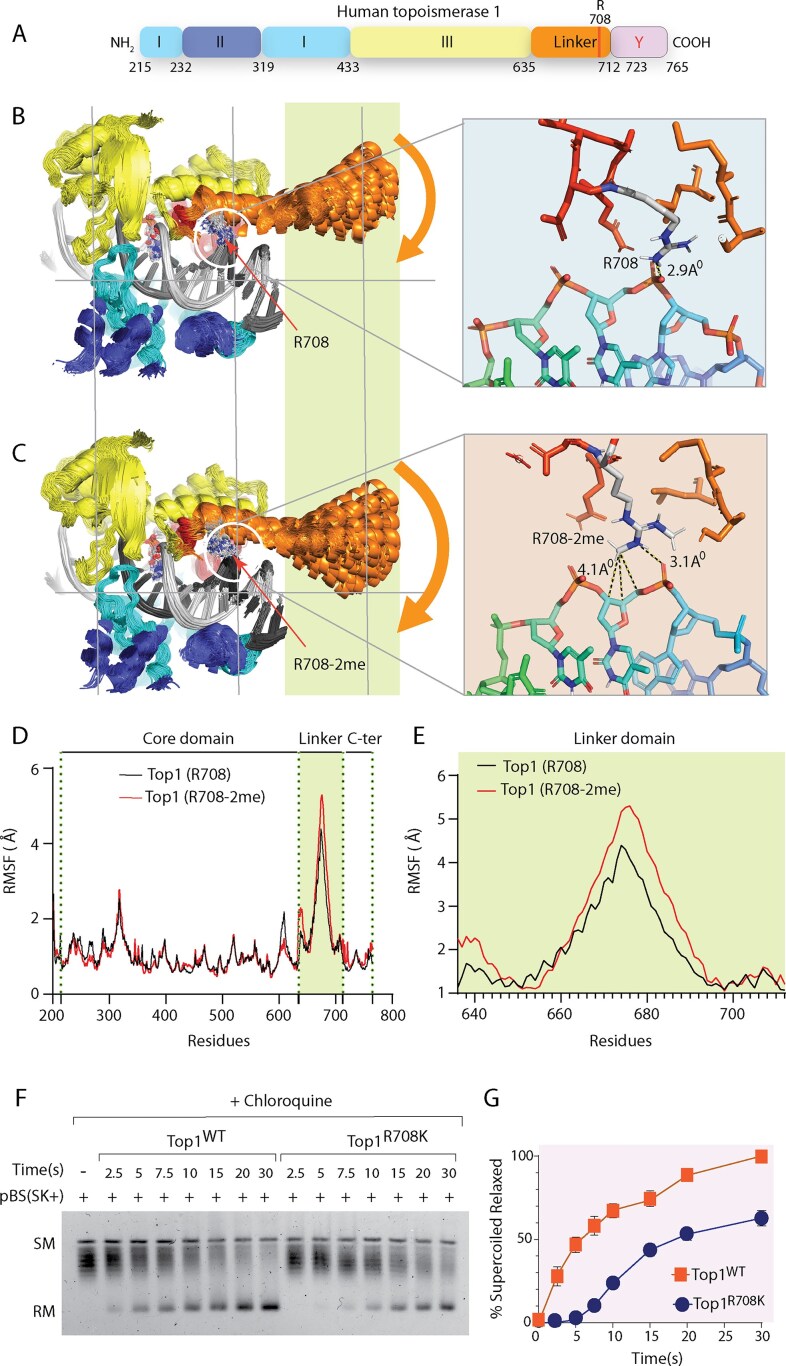
Arginine methylation facilitates DNA strand rotation. (**A**) Schematic domain organization of human Top1. Core domain includes subdomain I (sky blue) (residues 215–232, 320–433), subdomain II (glaucous) (residues 233–319), and subdomain III (yellow) (residues 434–635). The positions of the linker domain (orange) and the C-terminal domain (pink) are also indicated. (**B**) An equilibrium statistical ensemble of the DNA–Top1 complex (unmethylated) microstates obtained from MD simulation. The range of mobility in the linker region is indicated by the curved arrow. Protein domains are colored according to the domain diagram (A). The intact strand of DNA is shown in dark gray and the scissile strand in light gray. The white circle highlights the contact of R^708^ with the intact DNA strand, which is further shown in the close-up view. The terminal NH_2_ groups of R^708^ form hydrogen bonds with the phosphate group located between the +7 and +8 positions. (**C**) An equilibrium statistical ensemble of the DNA–Top1 complex (methylated) microstates obtained from MD simulation. The range of mobility in the linker region is indicated by the curved arrow. Protein domains are colored according to the domain diagram (A). The intact strand of DNA is shown in dark gray and the scissile strand in light gray. The white circle highlights the contact of R^708^-2me with the intact DNA strand, which is further shown in the close-up view. In addition to forming a hydrogen bond, R^708^-2me forms hydrophobic contacts with the 3′, 4′, and 5′ C of the deoxyribose sugar moiety of the nucleoside at +7 positions. (**D**) Average per-residue root mean square fluctuation (RMSF) represented as a function of the residue number for the unmethylated (black line) and methylated (R^708^-2me) Top1 (red line). Subdomain boundaries are separated by vertical green dotted lines, and the linker domain is highlighted in mint green. All domains exhibited fluctuations below 2 Å, except the linker domain, which displayed higher mobility. (**E**) RMSF of the linker region, comparing unmethylated (R^708^) and methylated (R^708^-2me) Top1 states. Similar to panel (D), except the RMSF of the linker domain was plotted separately to emphasize differential mobility between the unmethylated and methylated (R^708^-2me) Top1–DNA complex. (**F**) Time-dependent plasmid DNA relaxation activity assay was performed to measure strand rotation in the presence of chloroquine (2 mg/ml) with the excess of EGFP-Top1^WT^ and EGFP-Top1^R708K^ enzymes (3 μg protein) and DNA (100 ng) at 30:1 ratio: Lane 1 pBS (SK+) DNA; lanes 2–8, equivalent to lane 1 but DNA was incubated with EGFP-Top1^WT^, lanes 9–15, identical to lane 1 but DNA was incubated with EGFP-Top1^R708K^ for indicated time points. (**G**) Graph representing the percentage of relaxed DNA over time. All reactions were stopped by adding SDS at a final concentration of 0.5% (w/v) and subsequently subjected to electrophoresis on a 0.7% agarose gel. Positions of SM, relaxed and nicked monomer (RM/NM) are denoted in the gel. All the experiments were performed three times and expressed as the mean ± SD.

Strand rotation is a critical step in Top1 catalytic cycle, where transient single-strand break permits controlled rotation to relieve supercoiling [1, 71]. To investigate whether methylation influences DNA strand rotation, we performed 100 ns MD simulations of methylated and unmethylated Top1–DNA complexes. The unmethylated Top1 interacted primarily with the DNA backbone, consistent with structural precedents [72, 73]. Notably, the core subdomains and C-terminal domain of Top1 primarily contact the central 10 bp around the DNA cleavage site, while the linker domain interacts specifically with the +6 to +10 bp region downstream of the cleavage site. Distinct residue-specific interactions were observed between the methylated and unmethylated Top1–DNA complexes (Fig. 4A–C). In the unmethylated complex, unique interactions involved the side chains of L^216^, R^349^, L^439^, T^501^, L^650^, L^700^, Q^704^, T^718^, and L^746^, as well as the main chain amides of Q^633^ and A^635^ (Supplementary Fig. S4C). In contrast, the methylated complex exhibited exclusive contacts with Q^318^, N^352^, H^632^, L^654^, N^722^, N^745^, and the main chain amide of D^533^ (Supplementary Fig. S4D). Notably, the methylated complex displayed a distinct set of protein–DNA contacts. Notably, R^708^ in the methylated form engaged both the phosphate backbone and sugar moiety at the +7 position, suggesting a more stable DNA interaction (Supplementary Fig. S4C and D).

RMSF analysis revealed similar flexibility across most domains, but enhanced mobility in the linker domain of methylated Top1 (Fig. 4D and E), which is crucial for the swiveling motion required for strand rotation (Fig. 4A–C). To explore whether this flexibility arises from intrinsic collective motions, we conducted normal mode analysis (NMA) using the ANM [55]. The NMA revealed that the linker domain undergoes collective, correlated movements that were significantly altered upon R^708^ methylation (Supplementary Fig. S4E and F). This modification likely enhances the dynamic coupling between the DNA-binding region and linker, facilitating more efficient strand rotation.

To experimentally validate these findings, we directly measured the strand rotation rate of EGFP-Top1^WT^ and the EGFP-Top1^R708K^ mutant using a chloroquine-assisted DNA relaxation assay with supercoiled plasmid. The DNA substrate pBS(SK+) used in the assay has a size of 2.9 kb, which corresponds to roughly 14 negative supercoils per DNA molecule. Thus, a 30-fold molar excess of the enzymes (EGFP-Top1^WT^ and EGFP-Top1^R708K^) is used in the assay to achieve conditions in which the reaction rates are independent of the association or dissociation rates [74]. Under enzyme excess conditions, relaxed intermediates appeared at 2.5 s with EGFP-Top1^WT^, but only at 7.5 s with EGFP-Top1^R708K^ (Fig. 4F and G). This temporal delay confirms that R^708^ methylation accelerates strand rotation following cleavage complex (Top1cc) formation. Consistent with the “controlled rotation” model, in which multiple rotations can follow a single cleavage event, these findings indicate that methylation at R^708^ in the linker domain enhances the overall catalytic efficiency of Top1. Thus, arginine methylation likely modulates Top1 activity by influencing multiple steps in the catalytic cycle, including DNA cleavage and/or strand rotation.

### Arginine methylation at R^708^ and R^749^ regulates Top1–DNA cleavage complex stability

Next, we examined whether defective arginine methylation at R^708^ and R^749^ stabilizes the Top1cc by impairing DNA strand rotation. The delayed plasmid relaxation observed in methylation-deficient Top1, both in the presence and absence of CPT (Fig. 3H and J), likely stems from misalignment of the 5′-hydroxyl and 3′-tyrosine-linked DNA ends, thereby hindering religation and promoting Top1cc accumulation.

**Figure 5.**
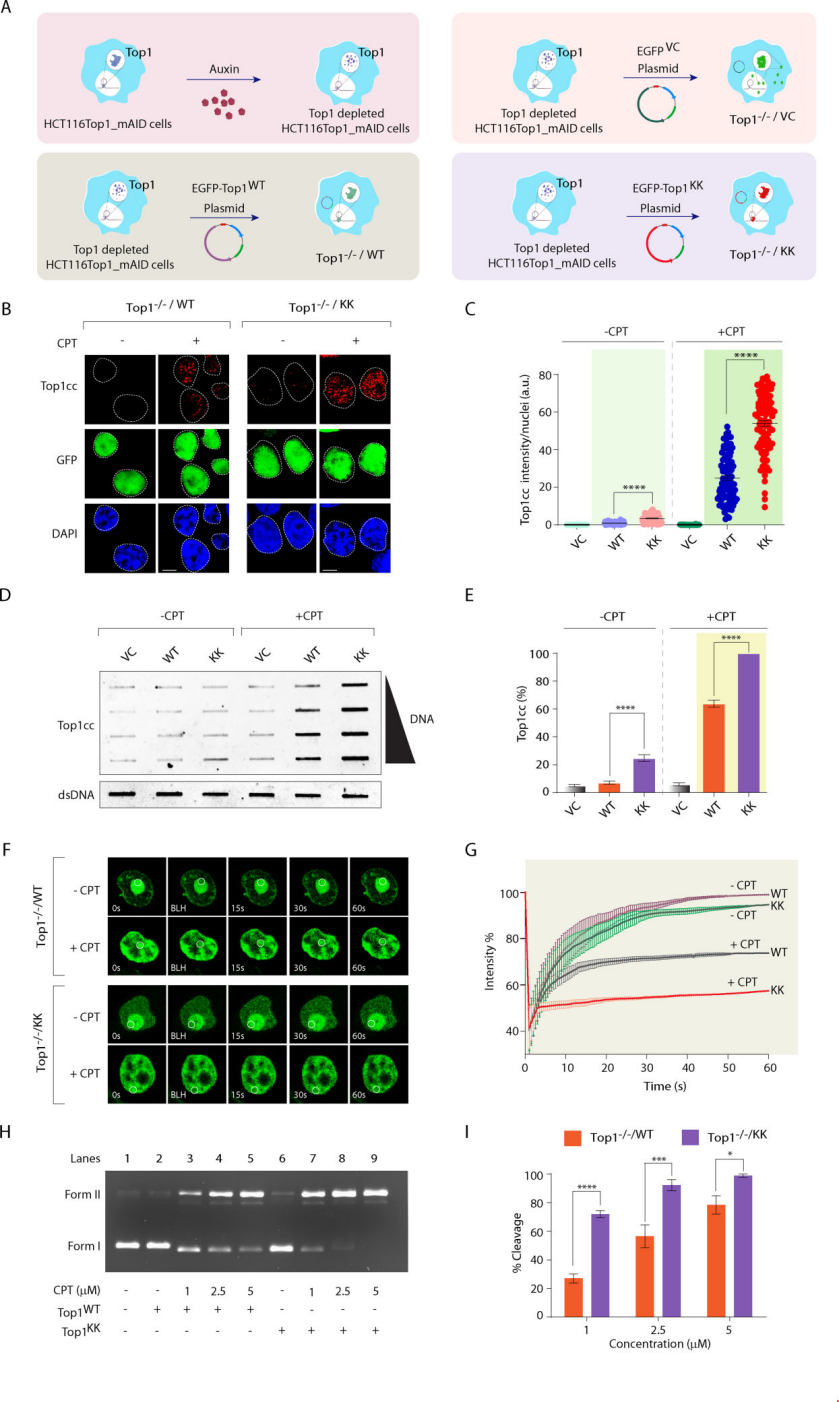
Abrogation of arginine methylation stabilizes Top1–cleavage complexes. (**A**) Schematic representation of the protocol followed to study the trapped Top1cc in Top1^−/−/VC^, Top1^−/−/WT^, and Top1^−/−/KK^ cells treated with or without CPT (5 µM, 3 h). (**B**) Representative images of confocal immunofluorescence microscopy show the CPT-induced Top1cc (red) and anti-GFP (green) as indicated. Cells were counterstained with DAPI to visualize nuclei (blue). Scale bar, 5 µm. (**C**) Measurement of Top1cc intensity per nucleus was calculated for 100–120 cells. Data are mean ± SEM, *n* = 3 biological replicates, ****P ≤ .0001 (*t*-test). (**D**) Top1cc was detected using the ICE (immunocomplex of enzyme) bioassay in Top1^−/−/VC^, Top1^−/−/WT^, and Top1^−/−/KK^ cells upon treatment with or without CPT (5 µM, 3 h). Increasing concentrations of genomic DNA (0.5, 1, 2, and 4 µg) were immunoblotted with anti-Top1cc antibody. The genomic DNA input was probed with anti-dsDNA antibody. (**E**) Densitometry analysis of the trapped Top1cc band intensity was quantified and expressed as fold increase with respect to genomic DNA input (error bars represent means ± SEM). *n* = 3 biological replicates, ****P ≤ .0001 (one-way ANOVA). (**F**) Representative images showing the FRAP of EGFP-Top1^WT^ and EGFP-Top1^KK^ ectopically expressing in auxin-treated HCT116TOP1_mAID cells and their response to CPT (1 µM), analyzed by confocal microscopy. A sub-nuclear ROI, indicated by a white circle, was bleached for 30 ms, followed by imaging at regular intervals of 0.5 s thereafter. Successive images captured for ∼60 s after photobleaching illustrate the fluorescence recovery within the bleached regions. (**G**) Quantification of FRAP data showing mean curves of EGFP-Top1^WT^ and EGFP-Top1^KK^ in the presence and absence of CPT. Error bars represent mean ± SD (*n* = 3). (**H**) Top1-mediated plasmid DNA cleavage assay. Representative gel showing Top1-mediated plasmid DNA cleavage in the presence of CPT. Lane 1, pBS (SK+) supercoiled DNA (300 ng). Lane 2, same as lane 1 but incubated with immunopurified EGFP-Top1^WT^ (0.2 μg protein), Lane 3–5, equivalent to lane 1 but incubated with equal amounts of EGFP-Top1^WT^ at the indicated concentrations of CPT. Lane 6, similar to lane 1 but incubated with (0.2 μg protein) EGFP-Top1^KK^, Lane 7–9, equivalent to lane 1 but incubated with equal amounts of EGFP-Top1^KK^ at the indicated concentrations of CPT at 37°C for 30 min. Positions of supercoiled substrate (Form I) and nicked monomers (Form II) are indicated. (**I**) Graph representing the percentage of nicked DNA (% cleavage) over time. Error bars represent mean ± SEM. *n* = 3 biological replicates. *P ≤ .05, ***P ≤ .001, ****P ≤ .0001 (two-way ANOVA). Asterisks denote statistically significant differences. a.u. arbitrary unit.

To directly assess the accumulation of Top1cc in cells lacking Top1 arginine methylation, we employed three independent assays. First, we complemented endogenous Top1-depleted HCT116Top1_mAID (Top1^−/−^) cells [39, 40] with either an empty vector (Top1^−/−/VC^), EGFP-Top1^WT^ (Top1^−/−/WT^), or arginine methylation mutant EGFP-Top1^KK^ (Top1^−/−/KK^). Successful expression of Top1 variants was confirmed by western blotting (Fig. 5A and Supplementary Fig. S5A). Secondly, to test *in situ* Top1cc formation, we performed immunofluorescence microscopy using a Top1cc-specific antibody [57, 75]. Top1^−/−/KK^ cells showed Top1cc accumulation even without CPT, which markedly increased ∼2-fold upon CPT treatment compared to methylation-proficient Top1^−/−/WT^ cells (Fig. 5B and C), suggesting that defective DNA relaxation by EGFP-Top1^KK^ (Fig. 3H and J) enhances its trapping by CPT at the Top1–DNA covalent interface. As expected, no Top1cc signal was detected in vector-only cells (Top1^−/−/VC^), irrespective of CPT treatment (Supplementary Fig. S5B).

Third, to directly measure the *in vivo* trapping of Top1cc in cells we used ICE assay [58]. Consistent with the immunofluorescence results (Fig. 5B and C), we detected an elevated Top1cc level in Top1^−/−/KK^ cells under basal conditions, which was increased ∼2-fold upon CPT exposure relative to Top1^−/−/WT^ cells (Fig. 5D and E). Together, these results suggest that Top1 arginine methylation facilitates DNA strand rotation, thereby favoring cleavage-religation equilibrium toward religation.

To further investigate whether the increased trapping of Top1^KK^ (Fig. 5B and D) results from defects in its nuclear dynamics [3], we performed FRAP using EGFP-tagged human Top1 variants (WT and KK) ectopically expressed in Top1^−/−^ (HCT116Top1_mAID) cells. We further utilized mathematical modeling to estimate the immobile fractions of Top1 (bound Top1cc) in the presence and absence of CPT. In the absence of CPT, FRAP analysis showed that EGFP-Top1^WT^ exists as a predominantly mobile population (∼98%–99%) with a minor immobile fraction (∼1%–2%) indicating dynamic nuclear mobility under steady-state conditions. Under similar conditions, EGFP-Top1^KK^ exhibited a small but repeated increase ∼4%–5% of immobile fraction even in the absence of CPT (Fig. 5F and G; No CPT). However, in the presence of CPT, Top1 is covalently trapped on the DNA which significantly blocks FRAP recovery by increasing ∼25%–30% of the EGFP-Top1^WT^ immobile population in the nucleoplasm (Fig. 5F and G). Notably, CPT treatment markedly increased (∼2 fold) the immobile fraction of EGFP-Top1^KK^ with an estimated bound fraction of ∼45%–50%, reflecting an extended residence time of Top1 on DNA (Fig. 5F and G; and kinetic fitting in Supplementary Fig. S5C and estimated immobile fractions in Supplementary Fig. S5D), suggesting altered Top1 dynamics in methylation mutant EGFP-Top1^KK^, favors CPT-induced Top1cc trapping (Fig. 5B–E). Under similar conditions, the catalytically inactive EGFP-Top1^Y723F^ mutant displayed no significant changes in mobility with or without CPT (Supplementary Fig. S5E and F).

This prompted us to directly assess the cleavage activity of methylation-proficient and -deficient Top1 variants using an *in vitro* plasmid DNA cleavage assay with immunoaffinity purified EGFP-Top1^WT^ and EGFP-Top1^KK^ proteins [45, 46]. In the presence of increasing concentrations of CPT, the cleavage reaction converts supercoiled closed circular DNA (Form I) into nicked circular DNA (Form II) by stabilizing the Top1–DNA cleavage complex, as shown in Fig. 5H. Quantitative analysis (Fig. 5I) reveals that the methylation-deficient EGFP-Top1^KK^ are stabilized on the cleaved DNA approximately two-fold more than EGFP-Top1^WT^, indicating that the absence of arginine methylation promotes CPT-induced Top1 trapping.

Further supporting the role of PRMT5-mediated arginine methylation in promoting DNA supercoil relaxation, we assessed Top1cc accumulation in PRMT5^−/−^ cells using confocal imaging and ICE assays. Supplementary Fig. S5G-J revealed a ∼2-fold increase in CPT-induced Top1cc in PRMT5^−/−^ compared to PRMT5^+/+^ cells, in keeping with Top1^−/−/KK^ cells (Fig. 5B and D). These findings underscore that PRMT5-mediated methylation of Top1 at R^708^ and R^749^ promotes linker domain flexibility, reducing Top1 residence time on DNA and facilitating efficient religation in the presence of CPT.

### PRMT5 inhibition enhances CPT-induced Top1cc formation: a rationale for combination therapy

Because PRMT5i are potent anticancer agents currently in clinical trials [29, 35] and effectively abrogate Top1 arginine methylation (Fig. 2G), we hypothesized that PRMT5i sensitizes cancer cells to Top1 poisons by enhancing Top1cc trapping. To test this, we examined the effect of the PRMT5i GSK3326595 on CPT-induced Top1cc formation.

**Figure 6.**
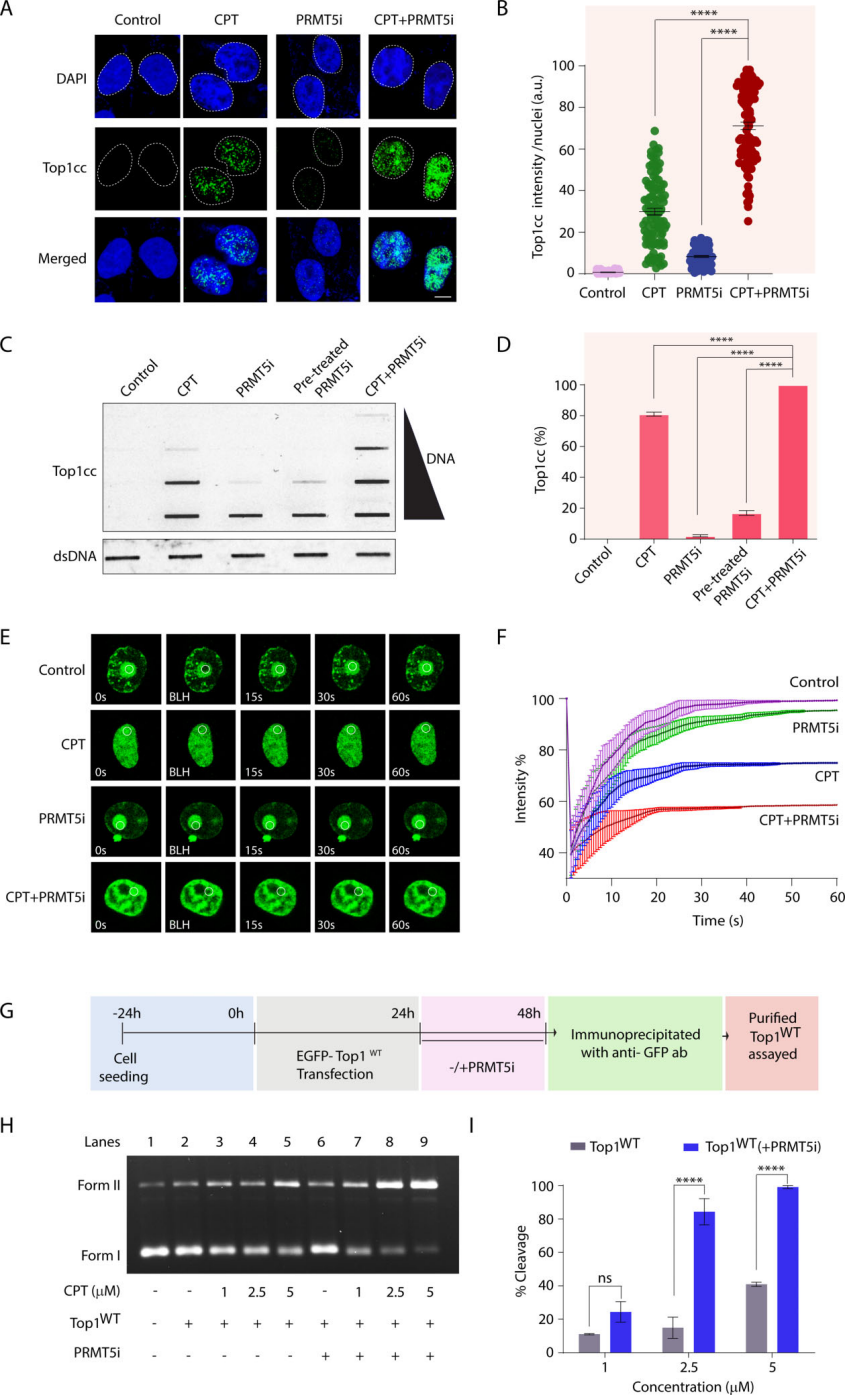
PRMT5i promotes the CPT-induced Top1cc. (**A**) Representative images of Top1cc (green) formation in MCF7 cells pre-treated with PRMT5i (5 µM, 24 h) or CPT (5 µM, 3 h), or both as indicated. Nuclei were stained with DAPI (blue). Scale bar, 5 µm. (**B**) Top1cc intensity per nucleus was obtained from immunofluorescence confocal microscopy for 100–120 cells. Data are mean ± SEM, ****P ≤ .0001 (*t*-test). (**C**) Trapped Top1cc detected using the ICE bioassay in MCF7 cells pre-treated with PRMT5i (5 µM, 24 h) or CPT (5 µM, 3 h) or both. Subsequently, increasing concentrations of genomic DNA (0.5, 1, 2, and 4 µg) were subjected to immunoblotting using an anti-Top1cc-specific antibody. The genomic DNA input was probed with anti-dsDNA antibody. (**D**) Densitometry analysis of the trapped Top1cc band intensity was quantified and expressed as fold increase with respect to genomic DNA input (error bars represent means ± SEM). *n* = 3 biological replicates, ****P ≤ .0001 (one-way ANOVA). (**E**) Representative images illustrating the FRAP of EGFP-Top1^WT^ expressing in auxin-treated HCT116TOP1_mAID cells pre-treated with PRMT5i (5 µM, 24 h), CPT (1 µM), or both. A sub-nuclear ROI, indicated by a circle, was bleached for 30 ms, followed by imaging at regular intervals of 0.5 s thereafter. Successive images captured for ∼60 s after photobleaching illustrate the fluorescence recovery within the bleached regions. (**F**) Quantification of FRAP data showing mean curves of EGFP-Top1^WT^ after pre-treatment with PRMT5i (5 µM, 24 h), CPT (1 µM), or in combination. Error bars represent mean ± SD (*n* = 3). (**G**) Schematic overview of the protocol used to study *in vitro* Top1-mediated plasmid DNA cleavage assay with immunoaffinity purified EGFP-Top1^WT^ from auxin-treated HCT116TOP1_mAID cells complemented with EGFP-Top1^WT^, in the presence and absence of PRMT5i treatment (5 µM, 24 h), using anti-GFP antibody. (**H**) Representative gel showing EGFP-Top1-mediated plasmid DNA cleavage in the presence of CPT. Lane 1: pBS (SK+) supercoiled plasmid DNA (300 ng). Lane 2: Lane 1 DNA incubated with untreated EGFP-Top1^WT^ (0.1 µg). Lanes 3–5: Same as Lane 2, incubated with increasing concentrations of CPT. Lane 6: Lane 1 DNA incubated with PRMT5i-treated EGFP-Top1^WT^. Lanes 7–9: Same as Lane 6, incubated with increasing concentrations of CPT. All reactions were carried out at 37 °C for 30 min. Positions of supercoiled (Form I) and nicked (Form II) plasmid DNA are indicated. (**I**) Quantification of CPT-induced DNA cleavage, shown as the percentage of nicked DNA (% cleavage). Error bars represent mean ± SEM, *n* = 3 biological replicates. ns non-significant (*P* > .05); ****P ≤ .0001 (two-way ANOVA). Asterisks denote statistically significant differences. a.u. arbitrary unit.

Immunofluorescence analysis in MCF7 cells revealed a significant (∼2-fold) increase in Top1cc upon combined PRMT5i and CPT treatment compared to either treatment alone (Fig. 6A and Supplementary Fig. S6A and B and quantification in Fig. 6B and Supplementary Fig. S6C). These results were corroborated by ICE bioassays, which also showed a ∼2-fold increase in Top1cc levels in cells co-treated with PRMT5i and CPT (Fig. 6C and quantification in Fig. 6D).

To assess how PRMT5i affects Top1 chromatin dynamics, we performed FRAP analysis in HCT116Top1_mAID cells expressing EGFP-Top1^WT^. PRMT5i reduced Top1 mobility (∼95% versus ∼99% mobile fraction), and co-treatment with CPT further decreased mobility to ∼58% compared to ∼75% with CPT alone (Fig. 6E and F; kinetic fitting in Supplementary Fig. S6D, estimated immobile fractions in Supplementary Fig. S6E), indicating increased Top1 retention on DNA. This mirrors observations in PRMT5^−/−^ and methylation-deficient Top1^KK^ cells, where impaired arginine methylation delays strand rotation and prolongs Top1–DNA interaction.

Finally, *in vitro*, plasmid cleavage assays confirmed that Top1 from PRMT5i-treated cells forms ∼2-fold more CPT-stabilized DNA cleavage complexes than untreated controls (Fig. 6G–I). Together, these findings establish that PRMT5i enhances Top1cc formation by delaying strand rotation, offering a compelling therapeutic strategy to potentiate Top1 poisons and overcome chemoresistance in cancer.

### Top1 deficient in arginine methylation forms persistent Top1cc, leading to DNA double-strand breaks and cell death

**Figure 7.**
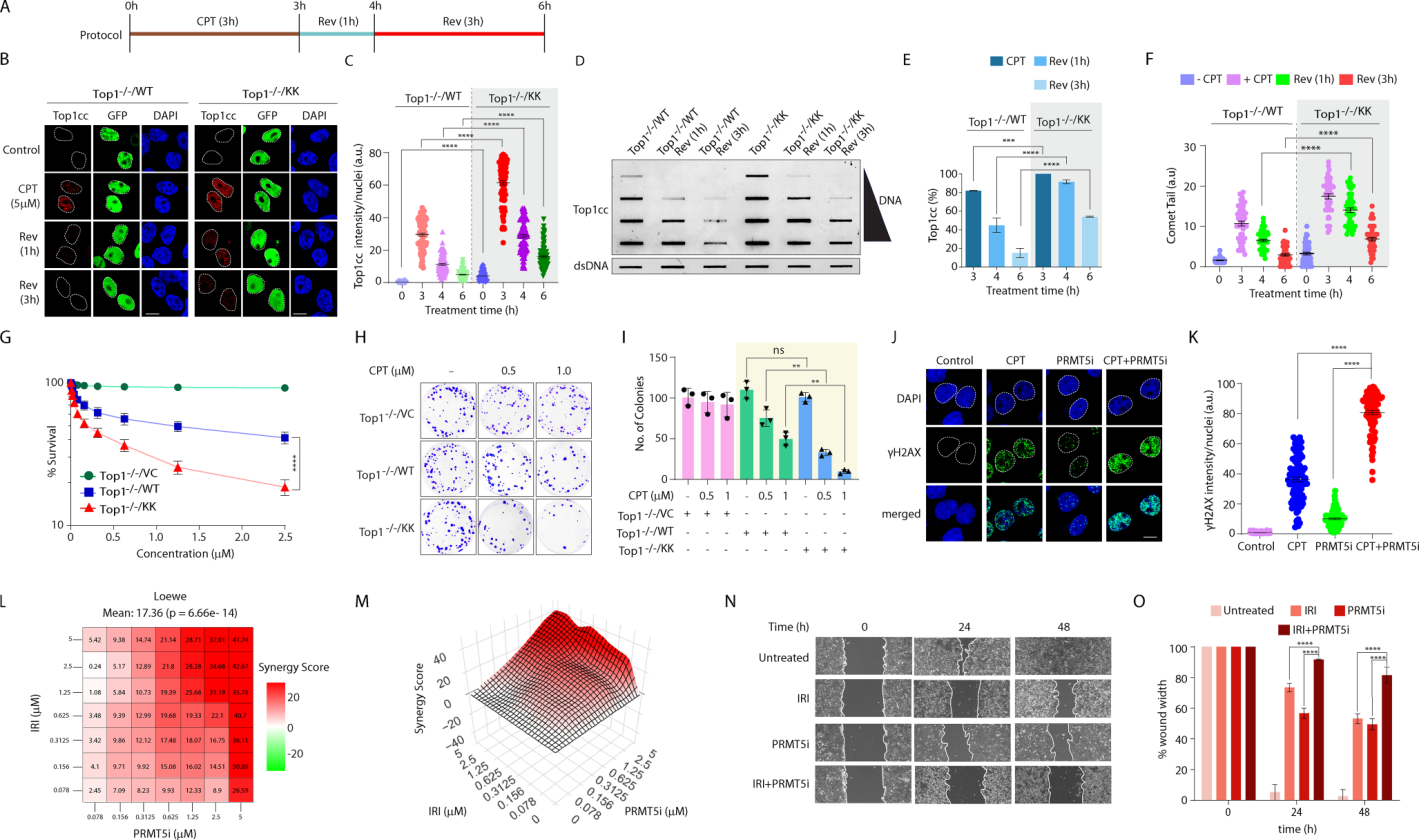
Top1 defective for arginine methylation makes persistent Top1cc, leading to DNA DSBs. (**A**) Schematic representation of the protocol followed for the detection of Top1cc after CPT removal. (**B**) Representative confocal microscopy images indicating the expression of EGFP-Top1 variants in Top1^−/−/WT^ and Top1^−/−/KK^, as detected through immunofluorescence staining using anti-GFP antibody (green) upon treatment with CPT (5 µM, 3 h). After CPT removal (Rev), cells were cultured in a drug-free medium for the indicated time points. Top1cc formation is shown in red, and nuclei were counterstained with DAPI (blue). Scale bar, 5 µm. (**C**) Measurement of Top1cc intensity per nucleus was calculated for 100–120 cells. Data are mean ± SEM, *n* = 3 biological replicates, ****P ≤ .0001 (*t*-test). (**D**) Trapped Top1cc was detected using the ICE bioassay in Top1^−/−/WT^ and Top1^−/−/KK^ cells upon treatment with CPT (5 µM, 3 h). Following CPT removal (Rev), cells were cultured for the indicated time points. Increasing concentrations of genomic DNA (0.5, 1, 2, and 4 µg) were immunoblotted with anti-Top1cc-specific antibody. The genomic DNA input was probed with anti-dsDNA antibody. (**E**) Densitometry analysis of the trapped Top1cc band intensity after the removal of CPT was quantified and expressed as fold increase with respect to genomic DNA input (error bars represent means ± SEM), ***P ≤ .001, ****P ≤ .0001 (one-way ANOVA). (**F**) Quantification of CPT-induced DNA strand breaks measured by comet assays in Top1^−/−/VC^, Top1^−/−/WT^, and Top1^−/−/KK^ cells upon treatment with CPT (5 µM, 3 h) followed by the removal of CPT as indicated. CPT-induced DNA strand breaks were calculated for 50 cells (mean ± SEM), ****P ≤ .0001 (*t*-test). (**G**) Cell survival curves of Top1^−/−/VC^, Top1^−/−/WT^, and Top1^−/−/KK^ cells. CPT-induced cytotoxicity (%) was calculated with respect to the untreated control. Error bars represent SD (*n* = 3), ****P ≤ .0001 (two-way ANOVA). (**H**) Representative images showing crystal violet colony formation in Top1^−/−VC^, Top1^−/−/WT^ and Top1^−/−/KK^ upon treatment with or without CPT as indicated. (**I**) Graphical representation showing the number of colonies. Error bars represent means ± SEM (*n* = 3), ns non-significant, **P ≤ .01 (two-way ANOVA). (**J**) Top1 inhibitor in combination with PRMT5i elevates DSBs. Confocal immunofluorescence microscopic analysis of γH2AX in MCF7 cells pretreated with PRMT5i (5 µM, 24 h), CPT (5 µM, 3 h) or both as indicated. γH2AX is shown in green. Nuclei were stained with DAPI (blue). Scale bar, 5 µm. (**K**) Quantification of CPT-induced γH2AX intensity per nucleus was calculated for 100 cells (calculated value ± SEM), ****P ≤ .0001 (*t*-test). (**L&M**) Top1 inhibitor synergizes with PRMT5i. The open-source Synergy Finder 2 was used to visualize the dose–response of the combination of Top1 inhibitor and PRMT5i in MCF7cells and to calculate Loewe synergy scores. (**N**) Representative images of the wound healing assay to measure the migration ability of MCF7 cells treated with PRMT5i (5 µM) or IRI (5 µM) or PRMT5i + IRI in a time-dependent manner (0, 24, and 48 h), magnification 10× . (**O**) The quantitative analysis of the wound healing assay. Error bars represent mean ± SEM, *n* = 3 biological replicates, ****P ≤ .0001 (two-way ANOVA). Asterisks denote statistically significant differences. a.u., arbitrary unit.

Loss of Top1 arginine methylation either through point mutation (Top1^KK^) (Fig. 2C), PRMT5 knockout (Fig. 2E), or PRMT5i (Fig. 2G) markedly increased trapped Top1cc after CPT treatment. Next, we investigated the role of Top1 arginine methylation in the persistence of CPT-induced Top1cc, given that under normal conditions, these complexes are efficiently resolved after CPT removal through religation of the nicked DNA strand [4]. To assess this, we compared the resolution kinetics of Top1cc between methylation-proficient (Top1^−/−/WT^) and methylation-deficient (Top1^−/−/KK^) cells after removal of CPT from the cultured media (Fig. 7A). Immunofluorescence microscopy showed that Top1cc signals in Top1^−/−/WT^ cells were significantly reduced by ∼80% within 1 h and nearly undetectable at 3 h post-CPT washout (Fig. 7B, panel CPT). In contrast, Top1^−/−/KK^ cells retained persistent Top1cc signals even after 1 or 3 h post-CPT washout, indicating slow reversal kinetics and prolonged trapping of Top1cc by ∼2-fold in Top1^−/−/KK^ compared to Top1^−/−/WT^ cells (Fig. 7B and C). ICE assays confirmed that Top1cc persisted in Top1^−/−/KK^ cells longer than in Top1^−/−/WT^ cells after 3 h post-CPT removal (Fig. 7D and E). Similar persistence of Top1cc was observed in PRMT5^−/−^ cells compared to PRMT5^+/+^ controls after CPT washout (Supplementary Fig. S7A–D), demonstrating that PRMT5-mediated arginine methylation promotes the catalytic activity of Top1.

To determine the consequences of persistent Top1cc, we evaluated DNA damage and cell survival in Top1^−/−/KK^ cells after CPT treatment. Comet assays revealed significantly more DNA strand breaks in Top1^−/−/KK^ than in Top1^−/−/WT^ cells, both during and after CPT treatment (Fig. 7F). Further, cell viability assays using auxin-inducible Top1 knockout (Top1^−/−^) cells complemented either EGFP-Top1^WT^ or EGFP-Top1^KK^ showed that methylation-deficient EGFP-Top1^−/−/KK^ conferred increased sensitivity to CPT, while vector control cells remained unresponsive to drug (Fig. 7G and Supplementary Fig. S7E). Colony formation assays further confirmed reduced survival in Top1^−/−/KK^ cells in the presence of CPT (Fig. 7H and I). Consistently, no significant difference in colony formation was observed in PRMT5^−/−^ cells expressing either EGFP-Top1^WT^ or EGFP-Top1^KK^ upon CPT treatment (Supplementary Fig. S7F and G), indicating that the methylation-deficient state renders them functionally indistinguishable with respect to proliferative growth.

To translate these findings pharmacologically, we treated MCF7 cells with the PRMT5i GSK3326595, either alone or in combination with CPT. Immunofluorescence revealed that combination treatment led to a ∼3-fold increase in γH2AX signal intensity, indicating significant DNA damage (Fig. 7J and K). Survival assays in MCF7 and HCT116 cells showed markedly reduced viability with PRMT5i + IRI (irinotecan, clinical derivative of CPT) co-treatment compared to monotherapy (Supplementary Fig. S7H and I). Synergy analysis using the Loewe model showed a high synergy score (17.36) for the PRMT5i + IRI combination (Fig. 7L and M). Furthermore, wound healing assays demonstrated impaired cell migration in the PRMT5i + IRI combination group, reinforcing the therapeutic benefit (Fig. 7N and O). In contrast, non-transformed breast epithelial MCF10A cells showed a markedly weaker response (∼15%–18% cell killing in MCF10A compare to ∼85%–87% cell killing in MCF7 cells under identical condition) to the combination treatment (PRMT5i + IRI, 5 μM), suggesting that the enhanced cytotoxicity of PRMT5i with IRI is preferentially selective toward cancer cells (Supplementary Fig. S7J). These findings provide strong mechanistic and functional evidence that combining PRMT5i with Top1 poisons offers a promising therapeutic strategy to enhance treatment efficacy and overcome resistance in cancers reliant on Top1 activity.

### PRMT5 inhibition enhances the antitumor activity of Top1 inhibitor to suppress tumor growth and metastasis in a preclinical TNBC model

To assess the therapeutic potential of combining PRMT5i and Top1 inhibitors *in vivo*, we evaluated this strategy in a 4T1 murine breast cancer model that recapitulates basal-like triple-negative breast cancer (B-TNBC), a subtype known for its aggressiveness, drug resistance, and high metastatic potential [62, 76]. To validate the relevance of this model for investigating Top1–PRMT5 functional interplay, we first examined whether Top1 and PRMT5 interact in 4T1-Chili-Luc cells. Co-IP analyses revealed a robust interaction between Top1 and PRMT5 under basal conditions, which remain unchanged following induction of DNA damage (Supplementary Fig. S8A), establishing that the Top1–PRMT5 axis is conserved in this model. We next evaluated the efficacy of combined Top1 and PRMT5i in 4T1 cells-Chili-Luc cells. Colony formation assays revealed a substantial reduction in clonogenic survival with IRI + PRMT5i (GSK3326595) treatment compared to monotherapies with IRI or PRMT5i (Supplementary Fig. S8B and C).

**Figure 8.**
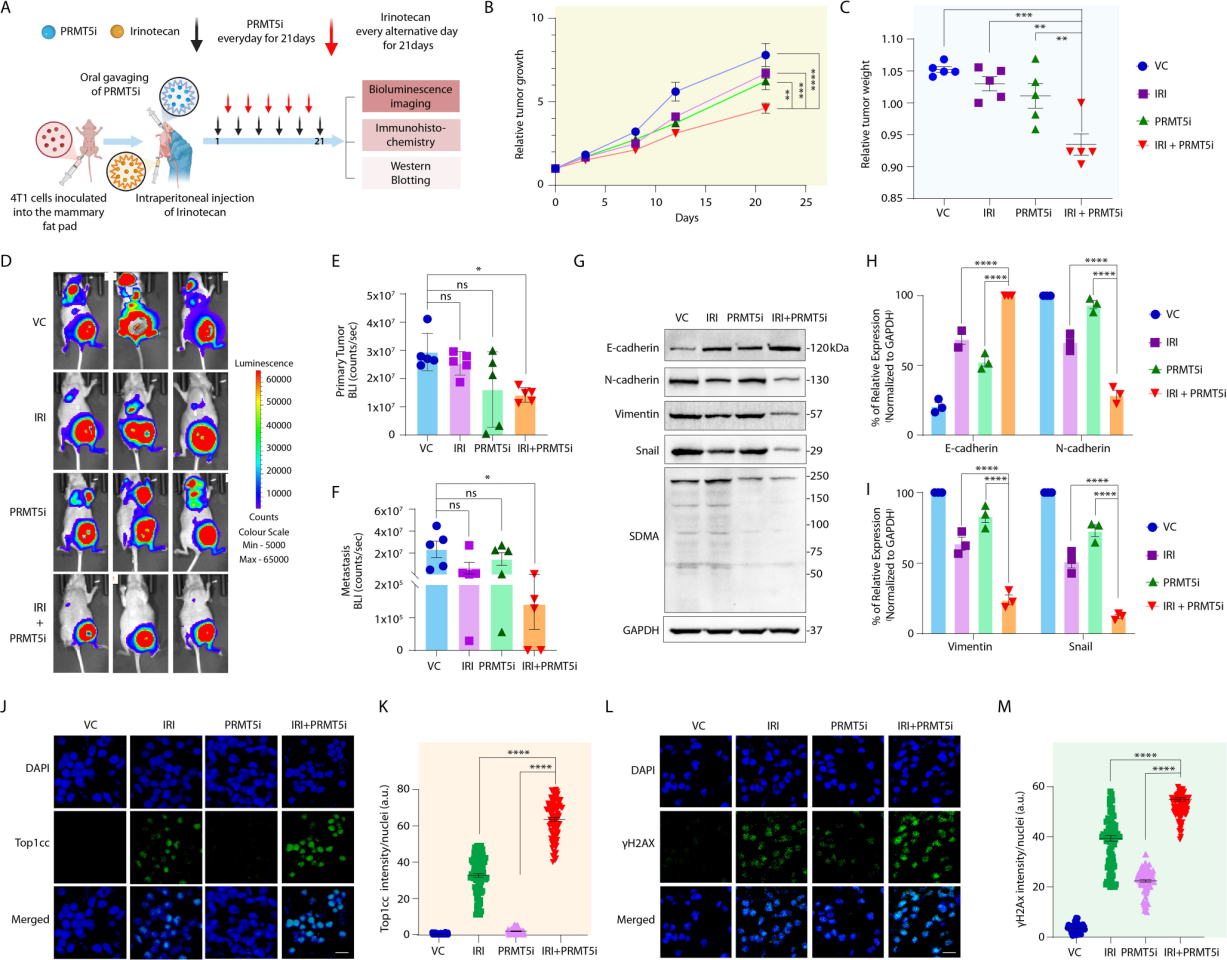
PRMT5i enhances the anti-tumor efficacy of Top1 inhibitor. (**A**) Schematic representation showing the experimental strategy of orthotopic inoculation of Td-Tomato, Luc tagged 4T1 cells into the mammary fat pad of nude Crl: CD1-Foxn1nu female mice treated with only IRI (5 mg/kg) every alternative day for 21 days, PRMT5i (20 mg/kg) daily, or IRI + PRMT5i for 21 days. (**B**) 4T1 tumor-bearing nude mice were treated as per the scheme. The line curves show relative tumor growth in VC, IRI alone, PRMT5i alone, and the combination of IRI and PRMT5i (IRI + PRMT5i). Data are mean ± SEM, *n* = 5, **P ≤ .01, ***P ≤ .001, ****P ≤ .0001 (two-way ANOVA). (**C**) Same as panel (B) except the graphical representation shows the relative mass of harvested tumors. Data are represented as mean ± SEM, *n* = 5, **P ≤ .01, ***P ≤ .001 (one-way ANOVA). (**D**) *In vivo* bioluminescence monitoring of primary tumor and distant metastatic sites 21 days after treatment commenced. The color scale indicated bioluminescence intensity (counts/s) emitted from each group. (**E**) Quantification of bioluminescence intensity calculated from the primary tumor site. Data are shown as mean ± SEM, *n* = 5, ns: non-significant (*P* > .05), *P ≤ .05 (one-way ANOVA). (**F**) Quantification of bioluminescence intensity calculated from the distant tumor site. Data are shown as mean ± SEM, *n* = 5, ns: non-significant (*P* > .05), *P ≤ 0.05 (one-way ANOVA). (**G**) Immunoblots showing the expression of epithelial-mesenchymal transition (EMT) markers E-cadherin, N-cadherin, vimentin, and snail in the tumor samples as indicated. Expression of SDMA was also checked. (**H&I**) Densitometry analysis of E-cadherin, N-cadherin, snail, and vimentin protein levels are shown. Data are presented as mean ± SEM (*n* = 3), ****P ≤ .0001 (two-way ANOVA). (**J**) Immunohistochemistry was carried out on treated tumor samples to detect the formation of Top1cc. Representative confocal images showing Top1cc (green) and nuclei stained with DAPI (blue). Scale bar, 8 µm. (**K**) Top1cc intensity was calculated from immunofluorescence confocal microscopy. Data are mean ± SEM, ****P ≤ .0001 (*t*-test). (**L**) Detection of γH2AX in treated tumor samples by immunohistochemistry. Representative confocal images showing γH2AX staining (green) and nuclei counterstained with DAPI (blue). Scale bar, 8 µm. (**M**) Quantitative analysis of γH2AX fluorescence intensity was calculated using confocal immunofluorescence microscopy. Results are expressed as mean ± SEM, ****P ≤ .0001 (*t*-test). Asterisks denote statistically significant differences. a.u. arbitrary unit.

For *in vivo* validation, 4T1-Chili-Luc cells were orthotopically implanted into the mammary fat pad of nude mice. Mice received IRI (5 mg/kg, i.p., alternate days), PRMT5i (20 mg/kg, oral, daily), or both for 21 days (Fig. 8A). Combination (IRI + PRMT5i) treatment markedly reduced relative tumor growth and weight (Fig. 8B and C) without noticeable toxicity (Supplementary Fig. S8D). Bioluminescence imaging on day 21 revealed significantly lower primary tumor growth and metastatic burden in the combination group compared to single-agent treatments or controls (Fig. 8D–F and Supplementary Fig. S8E).

Molecular analysis of excised tumors showed that combination therapy (IRI + PRMT5i) suppressed EMT, as evidenced by reduced Vimentin, N-cadherin, and Snail1, along with elevated E-cadherin (Fig. 8G and quantification in 8H–8I) [77, 78]. Decreased SDMA levels in PRMT5i-treated tumors confirmed effective PRMT5i *in vivo* (Fig. 8G). To further validate our findings, we performed H&E staining of lung sections revealed abundant metastatic foci in control tumor-bearing mice, whereas the combination treatment markedly reduced lung metastases compared with single-agent treatments (Supplementary Fig. S7F). Furthermore, Ki-67 staining demonstrated a significant drop in proliferation in the (IRI + PRMT5i) combination group [79] (Supplementary Fig. S8G and H).

Immunohistochemical staining of tumor sections revealed higher levels of Top1cc in the (IRI + PRMT5i) combination group (Fig. 8J and K), consistent with *in vitro* findings (Fig. 6A and B). This correlated with elevated γ-H2AX levels, indicating increased DNA DSBs (Fig. 8L and M). These effects likely stem from impaired Top1 activity due to the loss of PRMT5-mediated arginine methylation, prolonging Top1–DNA trapping.

Collectively, these findings demonstrate that PRMT5i potentiate the effects of Top1 inhibitors, enhancing Top1 trapping and increasing DNA damage, and suppress tumor growth and metastasis in aggressive B-TNBC. This combination represents a promising therapeutic strategy to overcome resistance and improve outcomes in Top1-targeted cancer therapies.

## Discussion

In this study, we show that PRMT5-mediated arginine methylation of Top1 at R^708^ and R^749^ enhances its catalytic activity by improving DNA supercoil relaxation, likely by modulating multiple steps of the reaction cycle, including DNA cleavage and strand rotation. Methylation-deficient Top1 (Top1^KK^) exhibits delayed DNA relaxation, impaired nuclear dynamics, and increased Top1cc accumulation that are independent of Top1–PRMT5 interaction but dependent on PRMT5’s catalytic activity. Loss of Top1 arginine methylation, either by point mutation, PRMT5 knockout, or pharmacological inhibition, significantly increases the persistence of trapped Top1cc following CPT treatment. Notably, combining PRMT5 with Top1 inhibitors enhances cancer cell killing across multiple cell types. In a preclinical TNBC model, this combination markedly increases DNA damage, suppresses tumor growth, and inhibits metastasis. Together, our findings reveal arginine methylation as a critical regulatory mechanism of Top1 activity and establish PRMT5i as a potent strategy to enhance Top1 poisoning, offering a promising therapeutic approach to overcome resistance in Top1-targeted cancer therapies.

PRMT5-mediated symmetric arginine methylation is a widespread PTM crucial for the DNA damage response, epigenetic regulation, and genome maintenance [25, 32]. While preserving the positive charge of arginine, methylation reduces hydrogen bond donors and increases hydrophobicity [35], thereby modulating protein stability, enzymatic activity, and protein–DNA interactions  [28, 29, 33]. Accordingly, PRMT5-mediated methylation enhances TDP1 catalytic efficiency [32], whereas PRMT1/3/6-mediated asymmetric dimethylation of TOP3β regulates its topoisomerase function [80]. Here, we identify Top1 as a novel PRMT5 substrate. PRMT5 symmetrically dimethylates Top1 at R^708^ and R^749^ residues (Fig. 2C), enhancing its DNA relaxation activity (Fig. 3 and Supplementary Fig. S3). We show that the NTD of PRMT5 directly binds the N-terminal region of Top1 (Fig. 1G and H), facilitating its methylation. Notably, deletion of the PRMT5 N-terminal TIM barrel domain which is critical for substrate recognition-abolishes this interaction (Fig. 1H), underscoring its essential role in Top1–PRMT5 complex formation. This observation is consistent with previous reports that the SH3 domain of c-Abl and the C-terminal domain of RNA polymerase II interact with the Top1 N-terminus to promote its DNA relaxation function [15, 65].

Mechanistically, the reduced DNA relaxation activity of the Top1 methylation mutant is attributed to persistent Top1cc and impaired strand rotation, not defective DNA binding. Top1 initiates catalysis by binding duplex DNA through its core and C-terminal domains, positioning Y^723^ for nucleophilic attack [73]. EMSAs showed no significant difference in DNA binding ability between EGFP-Top1^WT^ and EGFP-Top1^KK^, indicating that initial DNA recognition remains intact (Supplementary Fig. S4A and B).

Following cleavage, Top1 relaxes supercoiled DNA *via* “controlled strand rotation,” a process governed by downstream DNA interactions and modulated by the linker and specific α-helices [1, 81]. MD simulations (Fig. 4A–C) and chloroquine-assisted DNA relaxation assays revealed a pronounced decrease (three-fold) in DNA strand rotation efficiency in the Top1^R708K^ (Fig. 4F and G), highlighting the regulatory importance of R^708^ methylation. RMSF analysis further showed that methylation increases linker flexibility, supporting its role in enhancing the conformational dynamics required for efficient strand passage (Fig. 4D and E). This supports earlier findings that mutations like T^729^L in the linker region disrupt communication with the C-terminal domain, altering helical interactions, and impairing Top1 catalytic activity [13]. Notably, in the R^708^ methylated Top1–DNA complex, a unique hydrophobic interaction is observed with the sugar moiety of the intact strand (Fig. 4B and C; Supplementary Fig. S4C and D), in addition to canonical phosphate backbone contacts. This added interaction likely stabilizes the protein–DNA interface and facilitates efficient relaxation. Methylation at R^749^, located near the active site Tyr^723^, may support proper alignment of the 5′-cleaved DNA ends during religation, although further experiments are needed to confirm this function.

CPT poisons Top1, inhibit both strand rotation and religation, stabilizing Top1cc [82]. In HCT116Top1_mAID cells complemented with the EGFP-Top1^KK^ mutant, we observed a marked increase in CPT-induced Top1cc accumulation (Fig. 5B, D, and H), reflecting prolonged Top1–DNA residence time (Fig. 5F and G). This renders EGFP-Top1^KK^ more susceptible to CPT-induced trapping. Consistently, PRMT5 knockout or pharmacological inhibition similarly elevated Top1cc levels, as confirmed by immunofluorescence (Supplementary Fig. S5G and Fig. 6A), ICE assays (Supplementary Fig. S5I and Fig. 6C), and *in vitro* plasmid cleavage assays (Fig. 6H). These findings establish PRMT5-mediated arginine methylation as a key regulator of Top1 catalytic activity, promoting DNA supercoil relaxation through modulation of multiple steps in the catalytic cycle, including DNA cleavage, strand rotation, and CPT sensitivity.

Our findings demonstrate that arginine methylation-deficient Top1 shows prolonged retention of DNA after CPT withdrawal (Fig. 7A–F). Persistent Top1cc are cytotoxic as they impede replication and transcription, resulting in DNA DSBs [20, 41]. Consistently, Top1^−/−/KK^ cells exhibited elevated comet tail moments and increased γH2AX levels, both of which were further enhanced (∼2-fold) upon CPT treatment (Fig. 7F and J). These results underscore the critical role of PRMT5-mediated arginine methylation in resolving Top1cc and maintaining genome integrity.

PRMT5 is increasingly recognized as an oncogenic driver, with overexpression reported in breast, lung, colorectal, lymphoma, and glioma cancers [29, 83]. Accordingly, PRMT5i such as GSK3326595-currently in phase II trials for breast cancer and acute myeloid leukemia-are under clinical investigation [35, 84]. In our study, GSK3326595 cooperated with IRI to enhance cytotoxicity in breast (Supplementary Fig. S7H) and colorectal cancer cells (Supplementary Fig. S7I), primarily through the accumulation of persistent Top1cc resulting from impaired Top1 methylation. This cooperative effect was particularly evident in MCF7 cells, with a Loewe synergy score of 17.36 (Fig. 7L and M).

To assess *in vivo* efficacy, we employed the 4T1 mouse model of TNBC, which closely recapitulates human disease [62, 76]. The combination of PRMT5i and IRI significantly reduced tumor size and metastatic burden (Fig. 8B–F and Supplementary Fig. S8E), accompanied by increased DNA DSBs (Fig. 8L and M) and cancer cell death. Notably, tumors from the combination group showed elevated E-cadherin and reduced levels of EMT markers (N-cadherin, vimentin, Snail) (Fig. 8G), suggesting suppression of metastasis *via* modulation of EMT-related transcriptional or epigenetic programs [77, 78]. These findings align with prior reports linking PRMT5 to genome stability and DNA repair [30–32, 85, 86], including homologous recombination [30, 85, 87]. PRMT5i impairs Top1cc resolution and broader DNA repair responses, enhancing the cytotoxic effects of DNA-damaging agents like CPT and IRI.

In conclusion, we show that PRMT5 symmetrically dimethylates Top1 at R^708^ and R^749^—modifications essential for its catalytic function. Our data reveal a strong therapeutic rationale for combining PRMT5i with IRI, offering a promising strategy to overcome resistance where PARP inhibitors–IRI combinations have shown limited clinical success [88].

## Supplementary Material

gkag503_Supplemental_File

## Data Availability

All raw data and any additional information required to reanalyze the data reported in this paper are available from the lead contact upon request. The mass spectrometry proteomics data have been deposited to the ProteomeXchange Consortium via the PRIDE repository with dataset identifier PXD057645.
