# Supplementary material for: Arginine methylation of human DNA topoisomerase I by PRMT5 facilitates DNA relaxation
Source: Nucleic Acids Res. 2026 May 30;54(10):gkag503. doi: 10.1093/nar/gkag503 (PMC13221645; doi:10.1093/nar/gkag503)
Supplement: gkag503_Supplemental_File [file gkag503_supplemental_file.pdf]

Arginine Methylation of Human DNA Topoisomerase I by PRMT5 Facilitates DNA Relaxation

Saini Basu[1], Arpan Bhattacharyya[1,#], Muqtada Ali Khan[2,#], Uttam Pal[3], Srijita Paul Chowdhuri[1], Saumya Ranjan Satrusal[2, §], Laura Baranello[4], Dipak Datta[2, §] and Benu Brata Das[1*]

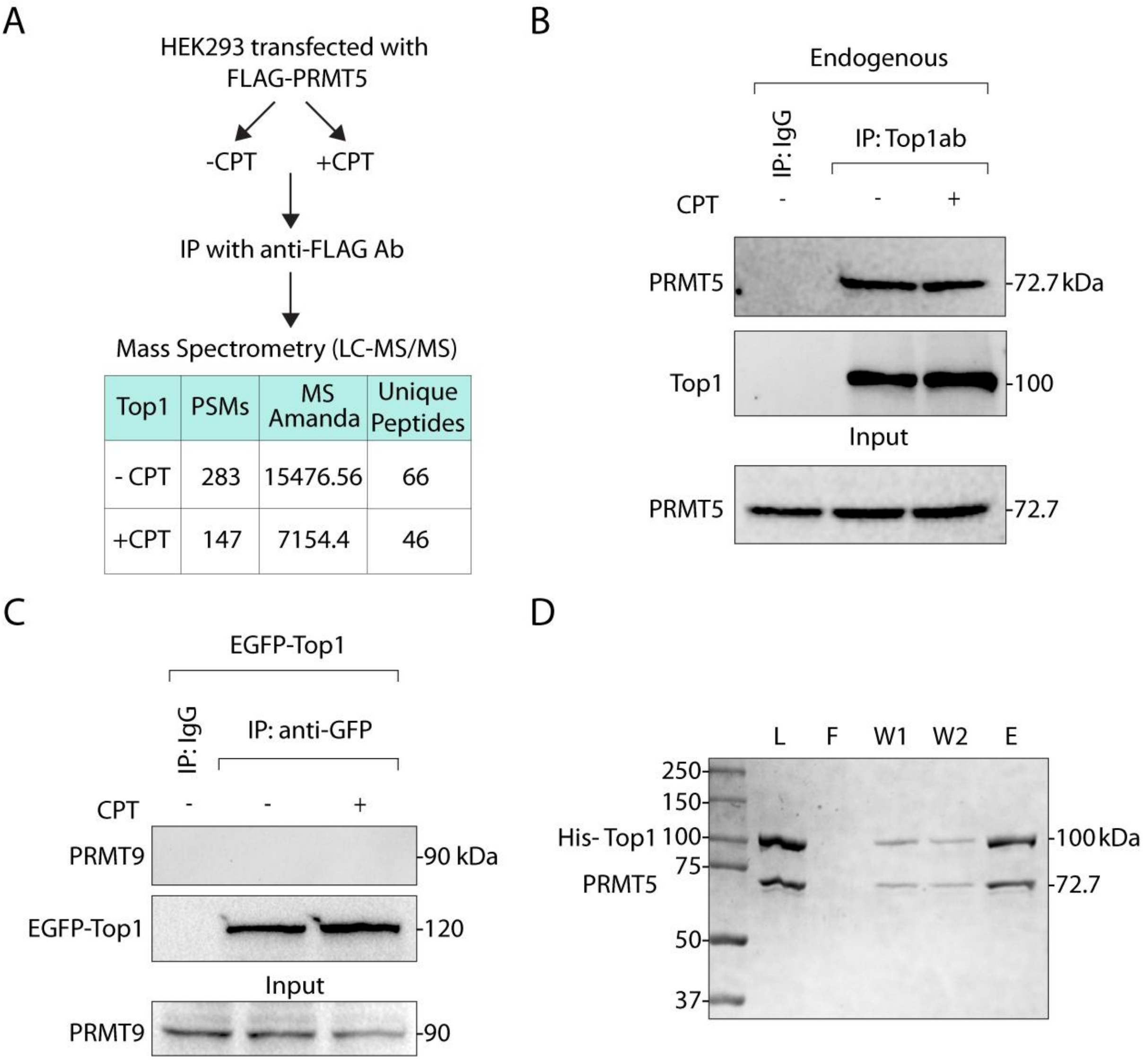

Figure S1: Top1 binds with PRMT5 but not with PRMT9. (**A**) FLAG-PRMT5 pull-down followed by LC-MS/MS analysis, ectopically expressed in HEK293 cells, reveals an interaction between PRMT5 and Top1, both with and without CPT treatment (5 µM, 3 h). FLAG-PRMT5 immunoprecipitation was performed using anti-FLAG antibody. Immunoprecipitation with control IgG antibody served as a control. Peptide-spectrum matches (PSMs), MS Amanda scores, and unique peptide counts for Top1 confirm this interaction irrespective of CPT. **(B)** Endogenous Top1 from HEK293 cells treated with or without CPT (5

Arginine Methylation of Human DNA Topoisomerase I by PRMT5 Facilitates DNA Relaxation

Saini Basu[1], Arpan Bhattacharyya[1,#], Muqtada Ali Khan[2,#], Uttam Pal[3], Srijita Paul Chowdhuri[1], Saumya Ranjan Satrusal[2, §], Laura Baranello[4], Dipak Datta[2, §] and Benu Brata Das[1*]

µM, 3 h) was immunoprecipitated using anti-Top1 antibody, and the immunocomplexes were analyzed by immunoblotting with anti-PRMT5 antibody. The blot was subsequently stripped and reprobed with anti-Top1 antibody to confirm uniform loading. Aliquots (10%) of the input lysates were immunoblotted to assess PRMT5 levels prior to immunoprecipitation. (**C**) HEK293 cells ectopically expressing EGFP-Top1 were treated with or without CPT (5 µM, 3 h), followed by anti-GFP immunoprecipitation. Western blot with anti-PRMT9 antibody showed no PRMT9 interaction with EGFP-Top1 immune complex. The blot was stripped and reprobed with anti-GFP to confirm equal loading. Aliquots (10%) of the input lysates were immunoblotted to assess PRMT9 levels prior to immunoprecipitation. **(D)** Ni^{2+}–NTA–agarose co-immobilization binding assay between His-tagged Top1 and untagged PRMT5. Lane L, His-Top1 and PRMT5 reconstituted complexes before loading onto Ni^{2+}–NTA–agarose beads. Lane F, flow-through containing unbound PRMT5. Lanes W1 and W2, excess unbound proteins after washing with 10 mM and 20 mM imidazole. Lane E, bound protein complexes eluted with 250 mM imidazole. Proteins were resolved by 10% SDS–PAGE and visualized by Coomassie Brilliant Blue staining.

Supplementary Information's

Arginine Methylation of Human DNA Topoisomerase I by PRMT5 Facilitates DNA Relaxation

Saini Basu[1], Arpan Bhattacharyya[1,#], Muqtada Ali Khan[2,#], Uttam Pal[3], Srijita Paul Chowdhuri[1], Saumya Ranjan Satrusal[2, §], Laura Baranello[4], Dipak Datta[2, §] and Benu Brata Das[1*]

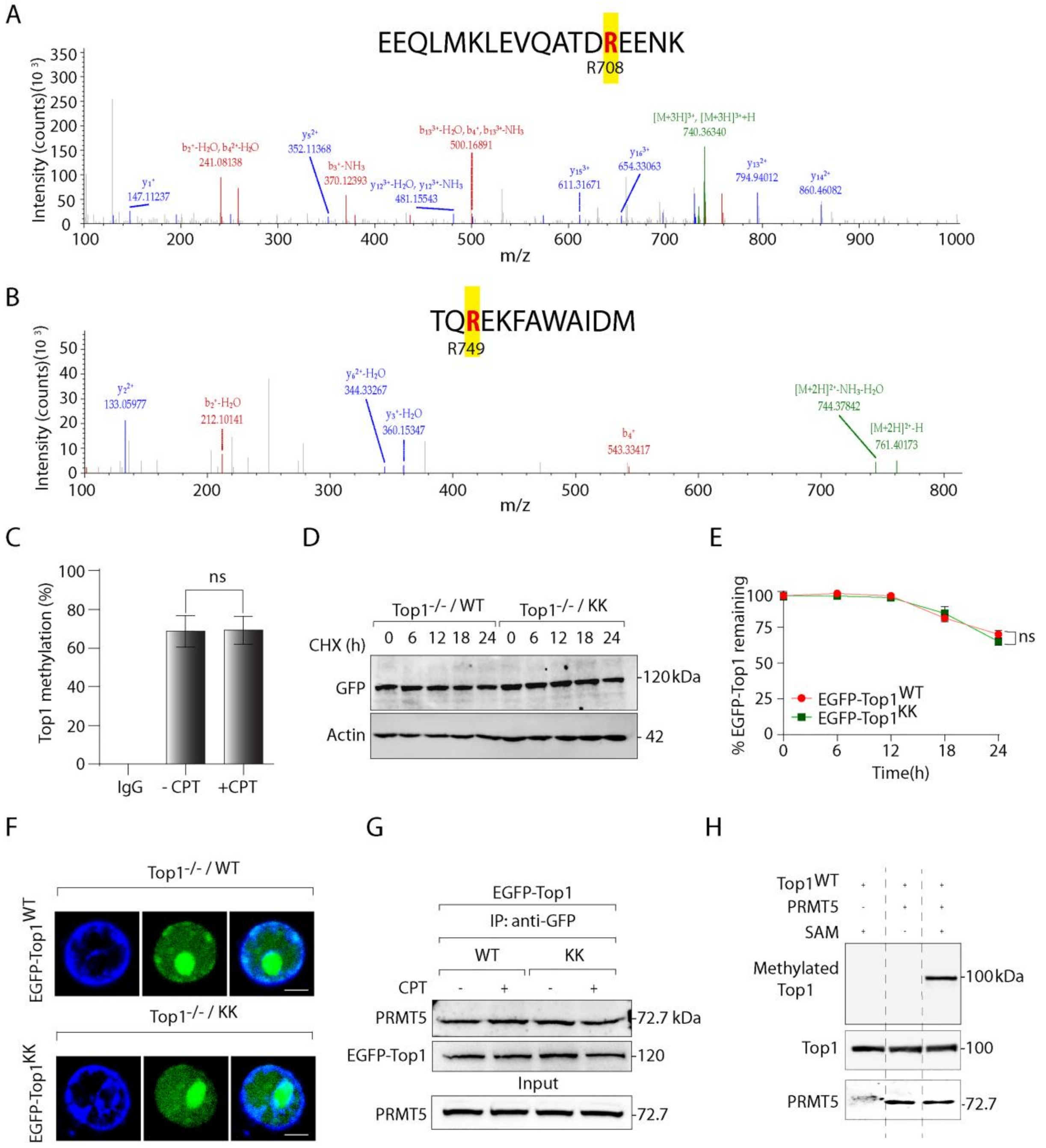

Figure S2: **Mass spectrometry profile of Top1 as detected by LC-MS/MS. (A-B)** MS/MS spectrum of the Top1 peptides (residues 695–712 aa and 747–758 aa) harbouring dimethylated arginine residues (R^{708} and R^{749}) highlighted in red. **(C)** Densitometry analysis showing symmetric arginine dimethylation level of EGFP-Top1^{WT} in the absence or presence of CPT

Saini Basu[1], Arpan Bhattacharyya[1,#], Muqtada Ali Khan[2,#], Uttam Pal[3], Srijita Paul Chowdhuri[1], Saumya Ranjan Satrusal[2, §], Laura Baranello[4], Dipak Datta[2, §] and Benu Brata Das[1*]

(5µM, 3h) was quantified and normalized to EGFP-Top1 and represented as fold change. Error bars represent mean ± SEM (n = 3). ns non-significant (P >0.05) (t-test). **(D-E)** Endogenous Top1-depleted HCT116 Top1_mAID ($Top1^{-/-}$) cells complemented with $EGFP\text{-}Top1^{WT}$ ($Top1^{-/-/WT}$) or $EGFP\text{-}Top1^{KK}$ ($Top1^{-/-/KK}$) were treated with cycloheximide (CHX) for the indicated time points (h) to examine Top1 protein stability. Protein levels were analyzed by western blotting (representative blots) (D), and the relative levels of $EGFP\text{-}Top1^{WT}$ and $EGFP\text{-}Top1^{KK}$ were quantified by densitometry normalized against actin (E). The remaining Top1 level was calculated relative to the level before CHX treatment. Error bars represent mean ± SEM (n = 3 biologically independent samples). ns non-significant (P >0.05) (two-way ANOVA). **(F)** Representative images showing the subcellular localization of EGFP-Top1 variants analyzed by live-cell confocal microscopy. Endogenous Top1-depleted HCT116 Top1_mAID ($Top1^{-/-}$) cells were transfected with $EGFP\text{-}Top1^{WT}$ or $EGFP\text{-}Top1^{KK}$ and imaged 48 h post-transfection. Both proteins localized predominantly to the nucleus, with no detectable differences in subcellular distribution between the two variants. Nuclei were stained with Hoechst 33342 (blue). Scale bar, 3µm. **(G)** To detect the interaction of PRMT5 with $EGFP\text{-}Top1^{KK}$, HEK293 cells ectopically expressing $EGFP\text{-}Top1^{WT}$ or $EGFP\text{-}Top1^{KK}$ were treated with or without CPT (5µM, 3h). Immunoprecipitation was performed using anti-GFP antibody. The immune complexes were blotted with anti-PRMT5-specific antibody. The same blot was stripped and reprobed using anti-GFP antibody to indicate equal loading. Aliquots (10%) of the input demonstrate the level of PRMT5 before immunoprecipitation. **(H)** In vitro methylation assay with FLAG-tagged PRMT5 immunoprecipitated from HEK293 cells using anti-FLAG antibody in the presence of unlabeled S-adenosylmethionine (SAM). The substrate used was recombinant His-tagged Top1. The reaction products were resolved by SDS-PAGE,

Arginine Methylation of Human DNA Topoisomerase I by PRMT5 Facilitates DNA Relaxation

Saini Basu[1], Arpan Bhattacharyya[1,#], Muqtada Ali Khan[2,#], Uttam Pal[3], Srijita Paul Chowdhuri[1], Saumya Ranjan Satrusal[2, §], Laura Baranello[4], Dipak Datta[2, §] and Benu Brata Das[1*]

and immunoblotted with anti-SDMA antibody. The blot was subsequently stripped and reprobed with anti-Top1 antibody. Asterisks denote statistically significant differences.

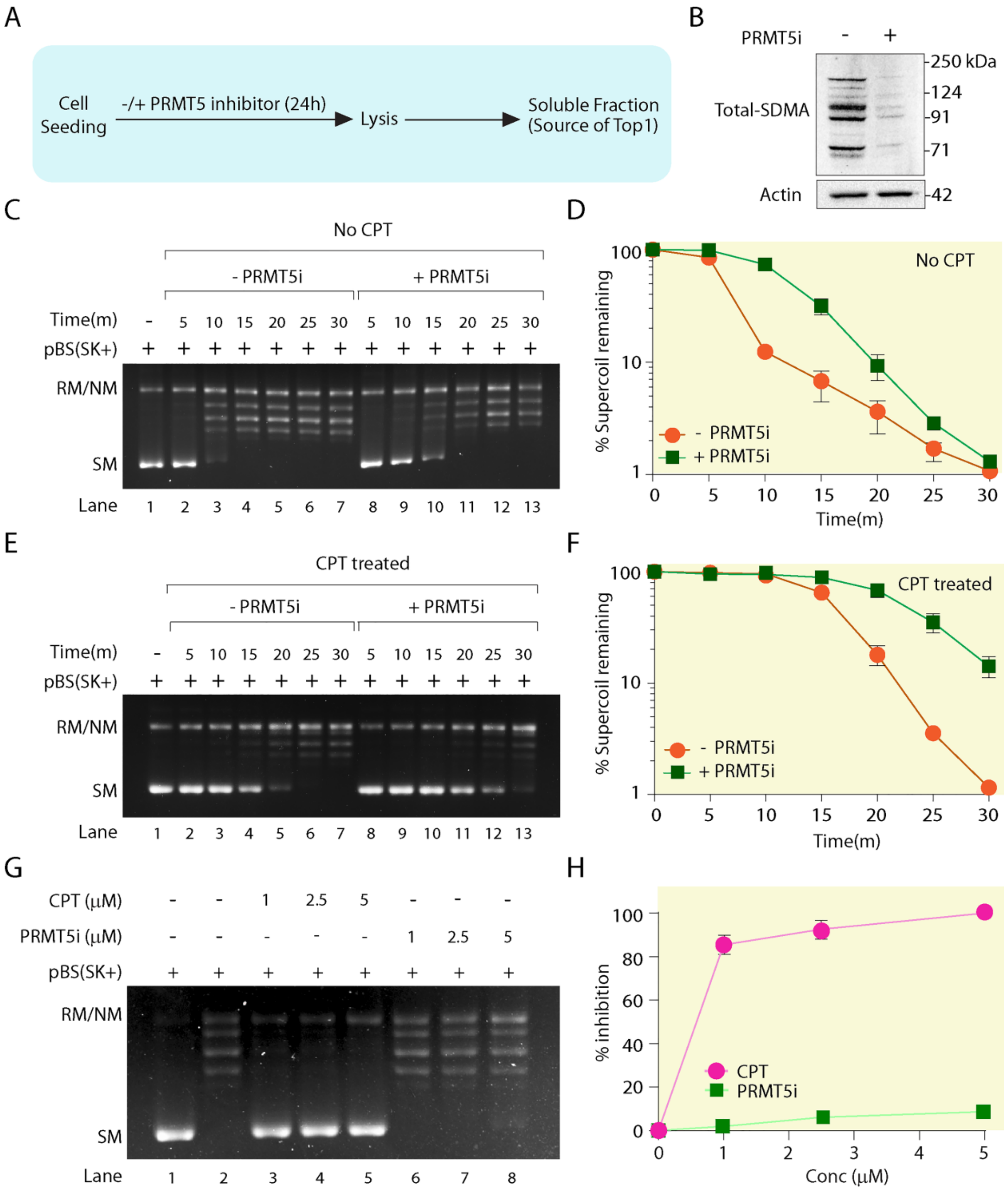

Arginine Methylation of Human DNA Topoisomerase I by PRMT5 Facilitates DNA Relaxation

Saini Basu[1], Arpan Bhattacharyya[1,#], Muqtada Ali Khan[2,#], Uttam Pal[3], Srijita Paul Chowdhuri[1], Saumya Ranjan Satrusal[2, §], Laura Baranello[4], Dipak Datta[2, §] and Benu Brata Das[1*]

Figure S3: PRMT5 regulates the catalytic activity of Top1 (A) Schematic representation showing the preparation of MCF7 whole cell lysates treated with or without PRMT5i (5µM, 24h) used as the source of endogenous Top1 for plasmid DNA relaxation activity assays. **(B)** Representative western blot showing the whole cell SDMA level. Actin served as a loading control. **(C)** Time dependent plasmid DNA relaxation activity assay was performed with or without PRMT5i treated (+PRMT5i) MCF7 cell lysates (each reaction volume contains 0.1 µg protein) in the absence of CPT: Lane 1 pBS (SK+) DNA (300 ng); lanes 2–7, equivalent to lane 1 but DNA was incubated with the PRMT5i untreated cell lysates for indicated time points; lanes 8-13, also equivalent to lane 1 but DNA was incubated with PRMT5i-treated cell lysates for indicated time points. **(D)** Graph representing the percentage of supercoiled plasmid DNA remaining over time. **(E)** Similar to (C), except the time-dependent plasmid DNA relaxation activity assay was performed in the presence of CPT **(F)**. Graphical depiction showing the percentage of supercoiled plasmid DNA remaining over time. **(G)** PRMT5i fails to inhibit the *in vitro* plasmid DNA relaxation activity of Top1 added during the relaxation assays. Lanes 1 pBS(SK+) DNA (300 ng); lane 2, identical to lane 1 but DNA was incubated with MCF7 cell lysates (each reaction volume contains 0.1 µg protein); lanes 3−5, same as lane 2 but MCF7 cell lysates were incubated with variable concentrations of CPT (as indicated); lanes 6−8, same as lane 2 but cell lysates were incubated with variable concentrations of PRMT5i (as indicated) at 37 °C for 30 min. **(H)** Quantitative representation for percentage relaxation inhibition (%) of endogenous Top1. All reactions were stopped by adding sodium dodecyl sulfate at a final concentration of 0.5% (w/v) and subsequently subjected to electrophoresis on a 1% agarose gel. Positions of supercoiled monomer (SM) and relaxed and nicked monomer (RL/NM) are denoted in the gel. All the experiments were performed three times and expressed as the mean ± SD.

Arginine Methylation of Human DNA Topoisomerase I by PRMT5 Facilitates DNA Relaxation

Saini Basu[1], Arpan Bhattacharyya[1,#], Muqtada Ali Khan[2,#], Uttam Pal[3], Srijita Paul Chowdhuri[1], Saumya Ranjan Satrusal[2, §], Laura Baranello[4], Dipak Datta[2, §] and Benu Brata Das[1*]

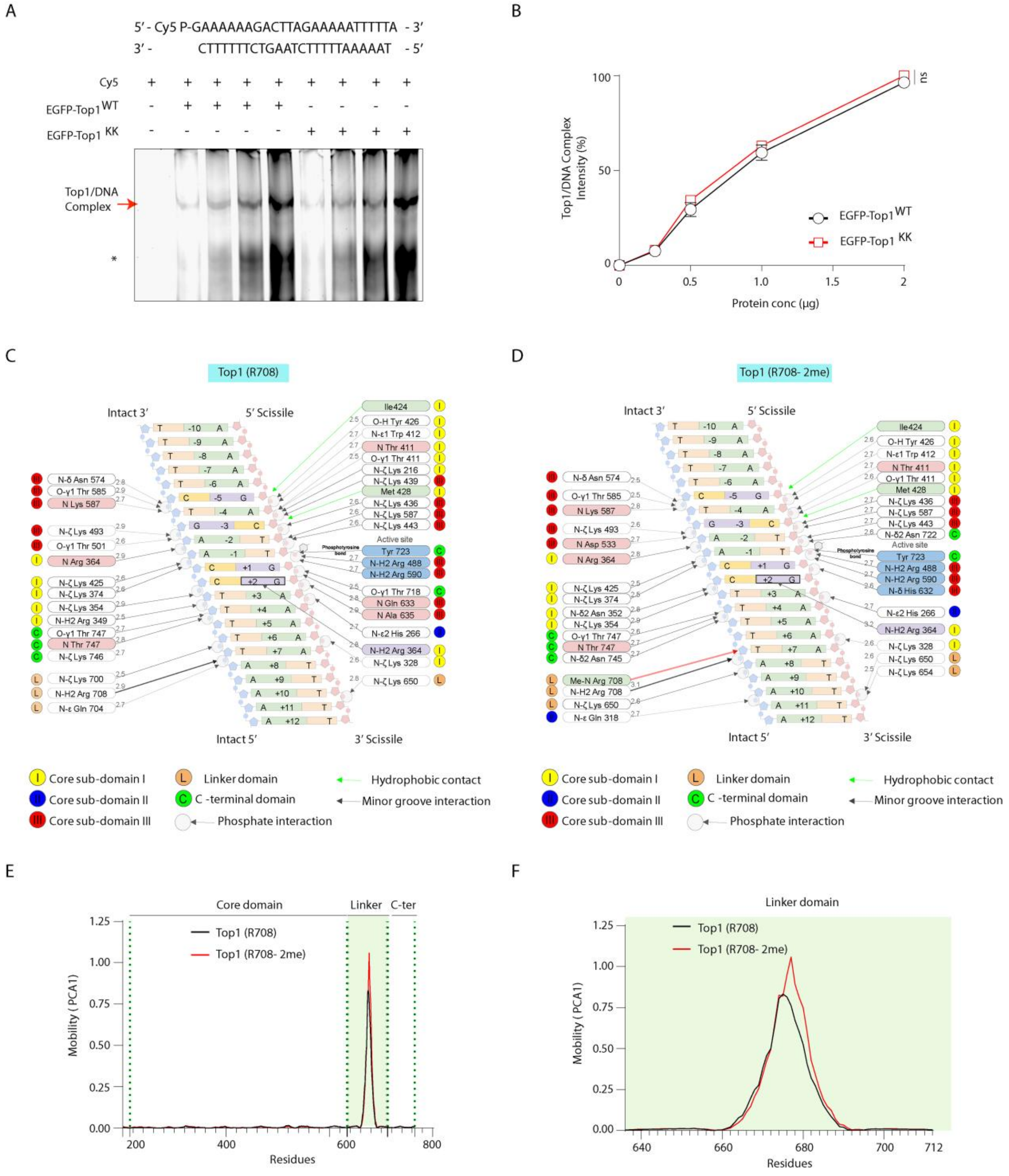

Figure S4: Arginine methylation enhances the mobility of the Top1 linker domain without altering its DNA-binding ability (A) Electrophoretic Mobility Shift Assay was performed in coincubations with Top1 variants (EGFP-Top1^{WT} and EGFP-Top1^{KK}) increasing concentration (0.25 – 2 µg proteins) and 5′-Cy5-end-labeled 25-mer duplex oligonucleotide (1 nM) (top

Arginine Methylation of Human DNA Topoisomerase I by PRMT5 Facilitates DNA Relaxation

Saini Basu[1], Arpan Bhattacharyya[1,#], Muqtada Ali Khan[2,#], Uttam Pal[3], Srijita Paul Chowdhuri[1], Saumya Ranjan Satrusal[2, §], Laura Baranello[4], Dipak Datta[2, §] and Benu Brata Das[1*]

panel). EGFP-Top1^{WT} and EGFP-Top1^{KK} were subjected to immunoaffinity purification using anti-GFP antibody. The native gel shift assay was carried out as described under "Materials and methods". The Top1-DNA complex is indicated by the red arrow. **(B)** The percent intensity of the Top1-DNA complex was quantified by a phosphor imager and plotted against the increasing protein concentrations. The binding profiles for EGFP-Top1^{WT} and EGFP-Top1^{KK} are indicated by black and red, respectively. All the experiments were performed three times and expressed as the mean ± SEM. ns non-significant (P >0.05) (two-way ANOVA). **(C)** Schematic representation of the protein-DNA interactions in the covalent complex of unmethylated human Top1 with DNA. Interactions between protein side chain and main chain atoms with DNA phosphate groups and minor-groove atoms closer than 3.5 Å are indicated. The protein-DNA contacts are limited almost exclusively to protein-phosphate interactions, and the protein contacts -5 to +10 bp around the cleavage site. The side chains of Lys^{216}, Arg^{349}, Lys^{439}, Thr^{501}, Lys^{650}, Gln^{704}, Thr^{718}, Lys^{746}, and the main chain amide nitrogen of Gln^{633}, Ala^{635} make contacts closer than 3.5 Å in this complex. **(D)** Schematic representation of the protein-DNA interactions in the covalent complex of methylated human Top1 (R^{708}-2me) with DNA. The side chains of Gln^{318}, Asn^{352}, His^{632}, Lys^{654}, Asn^{722}, Asn^{745}, and the main chain amide nitrogen of Asp^{533} make contacts closer than 3.5 Å in this complex. In addition, the methylated side chain of Arg^{708} makes an additional hydrophobic contact in this complex (indicated by solid red line) relative to the unmethylated complex. **(E)** Mobility profiles of the protein backbone in unmethylated (R^{708}) and methylated (R^{708}-2me) Top1 states obtained from ProDy ANM calculations using Normal Mode Wizard (VMD). The plot shows the per-residue fluctuations derived from the first non-trivial normal mode (PC1) out of the 10 calculated modes, which captures the dominant motion within the analyzed region. **(F)** Similar to (E) except the mobility profiles of the linker domain were plotted separately to emphasize differential mobility between the unmethylated and methylated Top1-DNA complex.

Supplementary Information's

Arginine Methylation of Human DNA Topoisomerase I by PRMT5 Facilitates DNA Relaxation

Saini Basu[1], Arpan Bhattacharyya[1,#], Muqtada Ali Khan[2,#], Uttam Pal[3], Srijita Paul Chowdhuri[1], Saumya Ranjan Satrusal[2, §], Laura Baranello[4], Dipak Datta[2, §] and Benu Brata Das[1*]

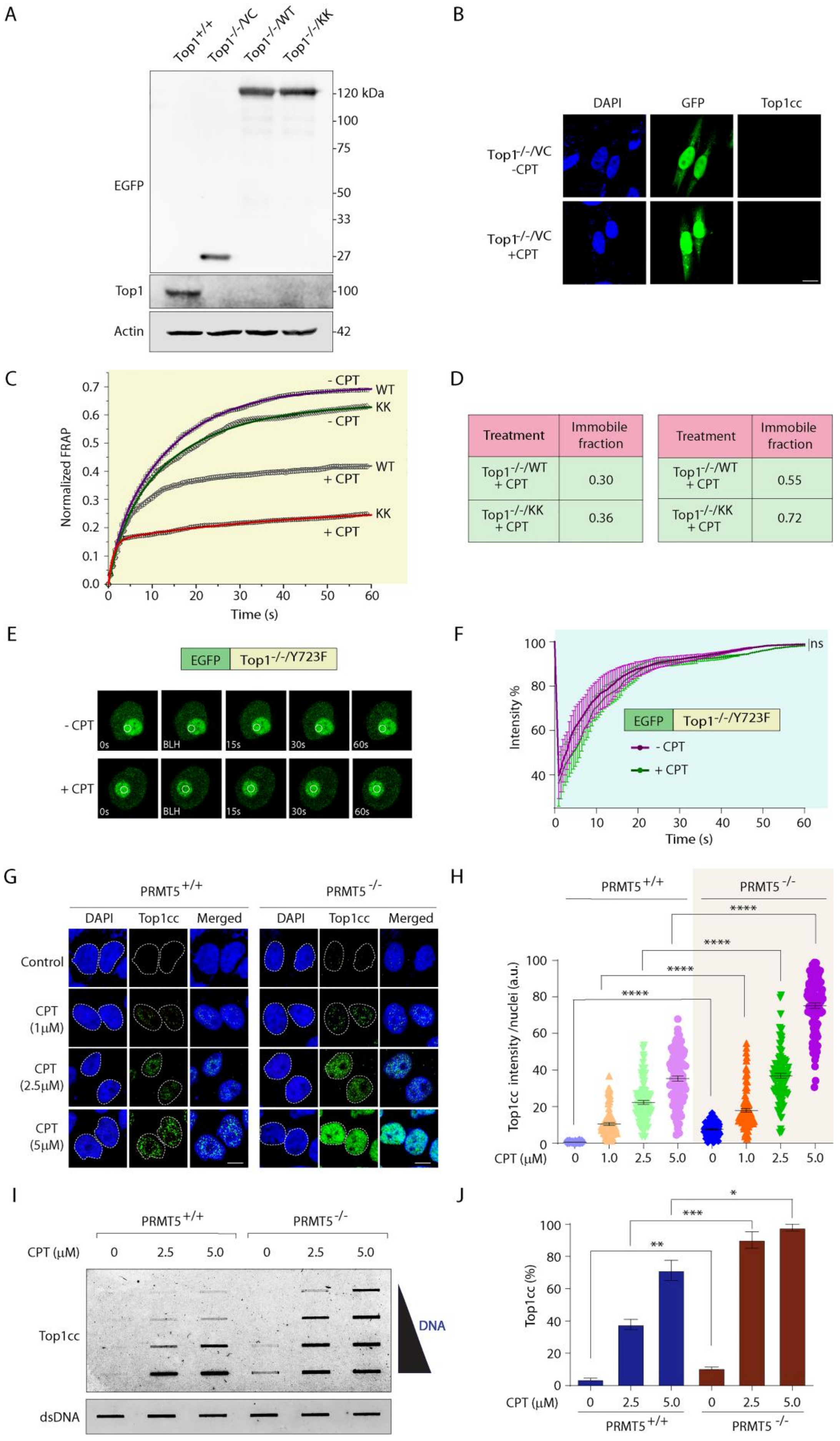

Arginine Methylation of Human DNA Topoisomerase I by PRMT5 Facilitates DNA Relaxation

Saini Basu[1], Arpan Bhattacharyya[1,#], Muqtada Ali Khan[2,#], Uttam Pal[3], Srijita Paul Chowdhuri[1], Saumya Ranjan Satrusal[2, §], Laura Baranello[4], Dipak Datta[2, §] and Benu Brata Das[1*]

Figure S5: Arginine methylation-deficient Top1 exhibits prolonged retention on DNA. (A) Representative western blot showing the complementation of auxin-inducible (HCT116TOP1_mAID) Top1 knockout ($Top1^{-/-}$) cells complemented either with EGFP empty vector ($Top1^{-/-/VC}$), EGFP-$Top1^{WT}$ ($Top1^{-/-/WT}$), or EGFP-$Top1^{KK}$ ($Top1^{-/-/KK}$) cells. Blots were probed with anti-GFP antibody to detect expression of EGFP-tagged proteins, and with anti-Top1 antibody to assess the efficiency of Top1 depletion. Actin served as a loading control. **(B)** Representative confocal microscopy images indicating the expression of EGFP empty vector ($Top1^{-/-/VC}$) in the presence and absence of CPT (5 µM). EGFP expression was detected through immunofluorescence staining using anti-GFP antibody (green). Top1cc formation is shown in red, and nuclei were counterstained with DAPI (blue). Scale bar, 10µm. **(C)** Normalized fluorescence intensities following photobleaching were fitted using Equation 1 (refer to Materials and Methods). Data points were collected up to 60 s for the following conditions: (a) EGFP-$Top1^{WT}$ (-CPT), (b) EGFP-$Top1^{WT}$ (+CPT), (c) EGFP-$Top1^{KK}$ (-CPT), and (d) EGFP-$Top1^{KK}$ (+ CPT). The corresponding fitted curves are represented by the solid line. **(D)** Normalized fluorescence intensities after photobleaching were fitted into Equation 1 (see 'Materials and Methods') (C) to quantify bound/immobile fractions of EGFP-$Top1^{WT}$ and EGFP-$Top1^{KK}$ in the absence or presence of Top1 poison (CPT). Quantification is based on mean FRAP data (n=3). **(E)** Representative images showing the FRAP of Top1 mutant EGFP-$Top1^{Y723F}$ ($Top1^{-/-/Y723F}$) ectopically expressing in Top1-depleted HCT116TOP1_mAID cells. Cells were treated with or without CPT (1 µM) and were analyzed by confocal microscopy. **(F)** Quantification of FRAP data showing mean curves of EGFP-$Top1^{Y723F}$ in the presence and absence of CPT. Error bars represent mean ± S.D. (n = 3). **(G)** Representative confocal images showing Top1cc (green) in PRMT5 $^{+/+}$ and $PRMT5^{-/-}$ MCF7 cells treated with varying concentrations of CPT (1, 2.5, and 5 µM). Nuclei were stained with DAPI (blue). Scale bar, 5µm. **(H)** Measurement of Top1cc intensity per nucleus was obtained from immunofluorescence confocal microscopy for 100–120 cells. Data are mean ± SEM, ****$P \leq 0.0001$ (t test). **(I)** Top1cc was detected using the ICE bioassay in PRMT5 $^{+/+}$ and $PRMT5^{-/-}$ MCF7 cells upon treatment with or without CPT (2.5 and 5 µM). Increasing concentrations of genomic DNA (0.5, 1, 2, and 4 µg) were immunoblotted with anti-Top1cc-specific antibody. The genomic DNA input was probed with anti-dsDNA antibody. **(J)**

Arginine Methylation of Human DNA Topoisomerase I by PRMT5 Facilitates DNA Relaxation

Saini Basu[1], Arpan Bhattacharyya[1,#], Muqtada Ali Khan[2,#], Uttam Pal[3], Srijita Paul Chowdhuri[1], Saumya Ranjan Satrusal[2, §], Laura Baranello[4], Dipak Datta[2, §] and Benu Brata Das[1*]

Densitometry analysis reveals the intensity of trapped Top1cc expressed as a fold increase relative to genomic DNA input (error bars represent means ± SEM). *P≤0.05, **P ≤0.01, ***P ≤0.001 (t-test). Asterisks denote statistically significant differences. a.u. arbitrary unit.

Arginine Methylation of Human DNA Topoisomerase I by PRMT5 Facilitates DNA Relaxation

Saini Basu[1], Arpan Bhattacharyya[1,#], Muqtada Ali Khan[2,#], Uttam Pal[3], Srijita Paul Chowdhuri[1], Saumya Ranjan Satrusal[2, §], Laura Baranello[4], Dipak Datta[2, §] and Benu Brata Das[1*]

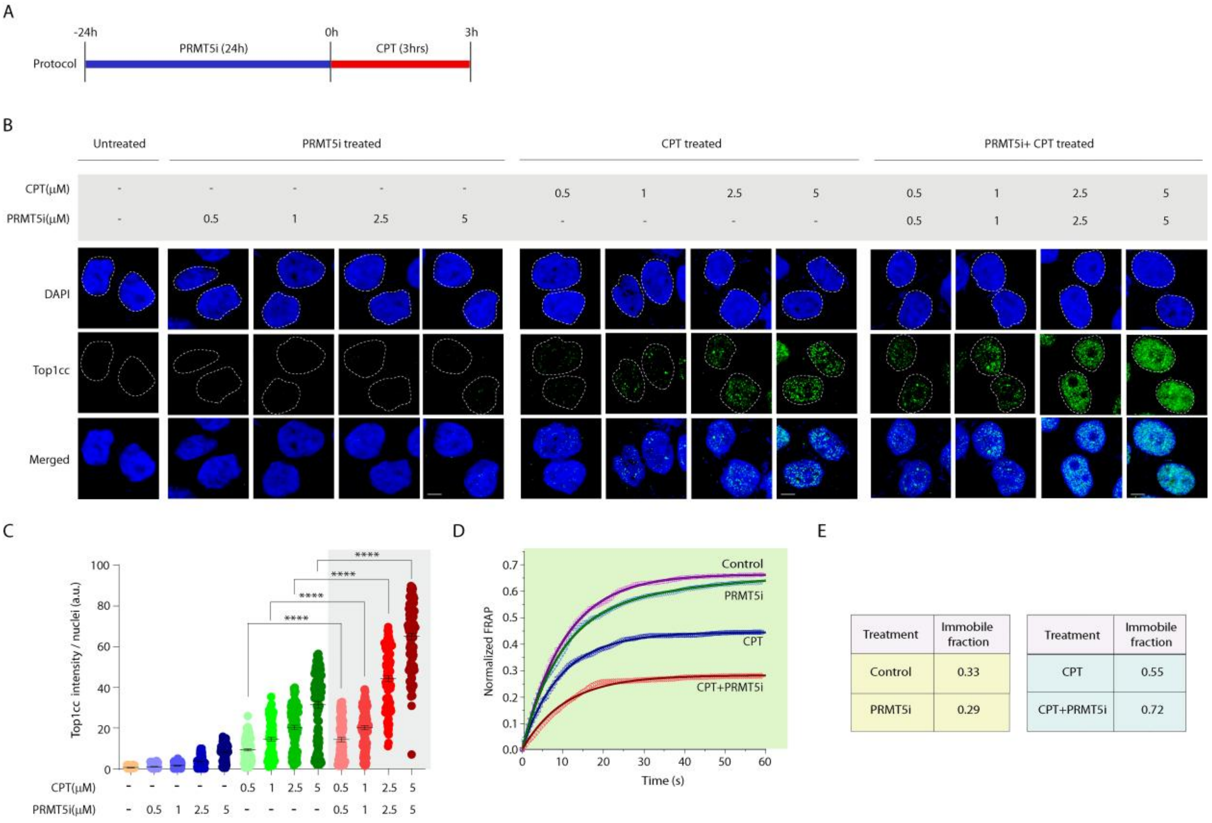

Figure S6: PRMT5i stabilizes CPT-induced Top1-cleavage complexes. (A) Schematic representation of the protocol followed for the detection of Top1cc in MCF7 cells pre-treated with varying concentrations of PRMT5i (0.5, 1, 2.5 & 5 µM, 24h) or CPT (0.5, 1, 2.5 & 5 µM, 3h) or combination of both (PRMT5i + CPT) as indicated. **(B)** Representative images of Top1cc (green) formation in MCF7 cells analyzed by confocal immunofluorescence microscopy. Nuclei were stained with DAPI (blue). Scale bar, 5µm. **(C)** Top1cc intensity per nucleus was obtained for 100-120 cells. Data are mean ± SEM, ****P≤0.0001 (t test). **(D)** Normalized fluorescence intensities of EGFP-Top1^{WT} following photobleaching were fitted using Equation 1 (refer to Materials and Methods). Data points were collected up to 60 seconds for the following conditions: (a) Control (-CPT), (b) PRMT5i (5µM, 24h), (c) CPT (1µM), and (d) CPT+PRMT5i-treated cells. The corresponding fitted curves are represented by the solid line. **(E)** The estimated values of the bound/immobile fraction of Top1 in the presence of indicated drugs after fitting in Equation 1 (refer to Materials and Methods). Asterisks denote statistically significant differences. Asterisks denote statistically significant differences. a.u. arbitrary unit.

Supplementary Information's

Arginine Methylation of Human DNA Topoisomerase I by PRMT5 Facilitates DNA Relaxation

Saini Basu[1], Arpan Bhattacharyya[1,#], Muqtada Ali Khan[2,#], Uttam Pal[3], Srijita Paul Chowdhuri[1], Saumya Ranjan Satrusal[2, §], Laura Baranello[4], Dipak Datta[2, §] and Benu Brata Das[1*]

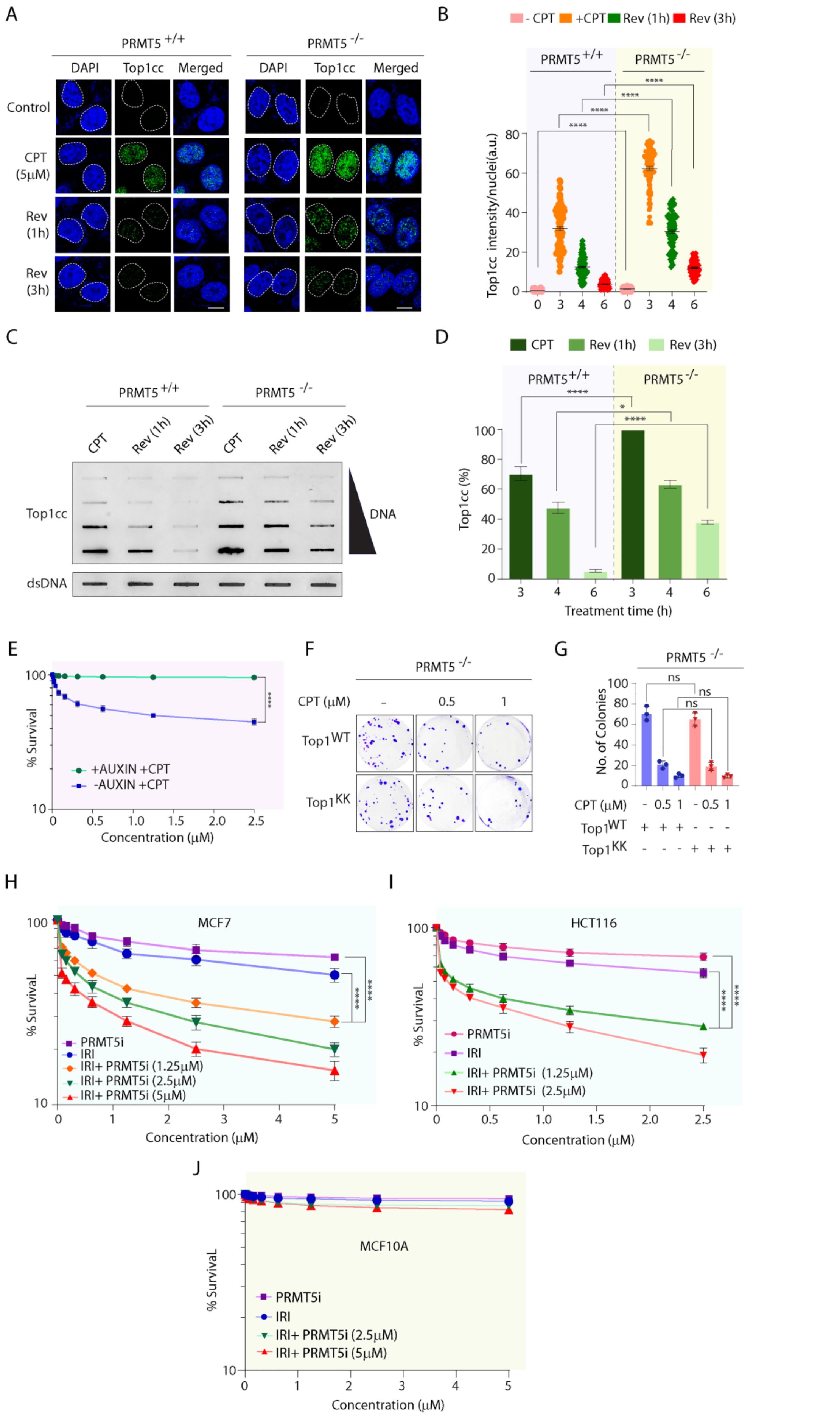

Arginine Methylation of Human DNA Topoisomerase I by PRMT5 Facilitates DNA Relaxation

Saini Basu[1], Arpan Bhattacharyya[1,#], Muqtada Ali Khan[2,#], Uttam Pal[3], Srijita Paul Chowdhuri[1], Saumya Ranjan Satrusal[2, §], Laura Baranello[4], Dipak Datta[2, §] and Benu Brata Das[1*]

Figure S7: Abrogation of Top1-arginine methylation leads to persistent Top1cc formation

(A) Representative confocal microscopy images showing the formation of CPT-induced Top1cc in PRMT5 $^{+/+}$ and PRMT5$^{-/-}$ MCF7 cells treated with CPT (5 µM, 3 h). Following CPT removal (Rev), cells were cultured in a drug-free medium for the indicated time points. Top1cc formation is shown in green, and nuclei were counterstained with DAPI (blue). Scale bar, 5µm. **(B)** Measurement of Top1cc intensity per nucleus was calculated for 100–120 cells (calculated value ±SEM), ****P≤0.0001 (t-test). **(C)** Trapped Top1cc was detected using the ICE bioassay in PRMT5 $^{+/+}$ and PRMT5$^{-/-}$ MCF7 cells treated with CPT (5µM, 3h). After CPT removal (Rev), cells were cultured for the indicated time points. Increasing concentrations of genomic DNA (0.5, 1, 2, and 4 µg) were immunoblotted with anti-Top1cc antibody. The genomic DNA input was probed with anti-dsDNA antibody. **(D)** Densitometry analysis of the trapped Top1cc band intensity after the removal of CPT was quantified and expressed as fold increase with respect to genomic DNA input (error bars represent means ± SEM), n=3 biological replicates. *P≤0.05, ****P≤0.0001 (one-way ANOVA). **(E)** Cell survival curves of Top1-depleted HCT116TOP1_mAID cells treated with or without Auxin. CPT-induced cytotoxicity (%) was calculated with respect to the untreated control. Error bars represent SD (n = 3), ****P≤0.0001 (two-way ANOVA). **(F)** Representative images showing crystal violet colony formation in PRMT5$^{-/-}$ cells complemented with EGFP-Top1^{WT} or EGFP-Top1^{KK} upon treatment with or without CPT as indicated. **(G)** Graphical representation showing the number of colonies. Error bars represent means ±SEM (n = 3), ns non-significant (two-way ANOVA). **(H)** Cell survival curves of MCF7 cells treated with PRMT5i or IRI or IRI pre-treated with different concentrations of PRMT5i. Cell cytotoxicity (%) of each group was calculated with respect to the untreated control. Each point corresponds to the mean ± S.D. (n=3), ****P≤0.0001 (two-way ANOVA). **(I)** Similar to (H) except the cell survival was performed in HCT116 cells. Cell cytotoxicity (%) of each group was calculated with respect to the untreated control. Each point corresponds to the mean ± S.D. (n=3), ****P≤0.0001 (two-way ANOVA). **(J)** Similar to (H) except the cell survival was performed in MCF10A cells. Cell cytotoxicity (%) of each group was calculated with respect to the untreated control. Each point corresponds to the mean ± S.D. (n=3). Asterisks denote statistically significant differences. a.u. arbitrary unit.

Supplementary Information's

Arginine Methylation of Human DNA Topoisomerase I by PRMT5 Facilitates DNA Relaxation

Saini Basu[1], Arpan Bhattacharyya[1,#], Muqtada Ali Khan[2,#], Uttam Pal[3], Srijita Paul Chowdhuri[1], Saumya Ranjan Satrusal[2, §], Laura Baranello[4], Dipak Datta[2, §] and Benu Brata Das[1*]

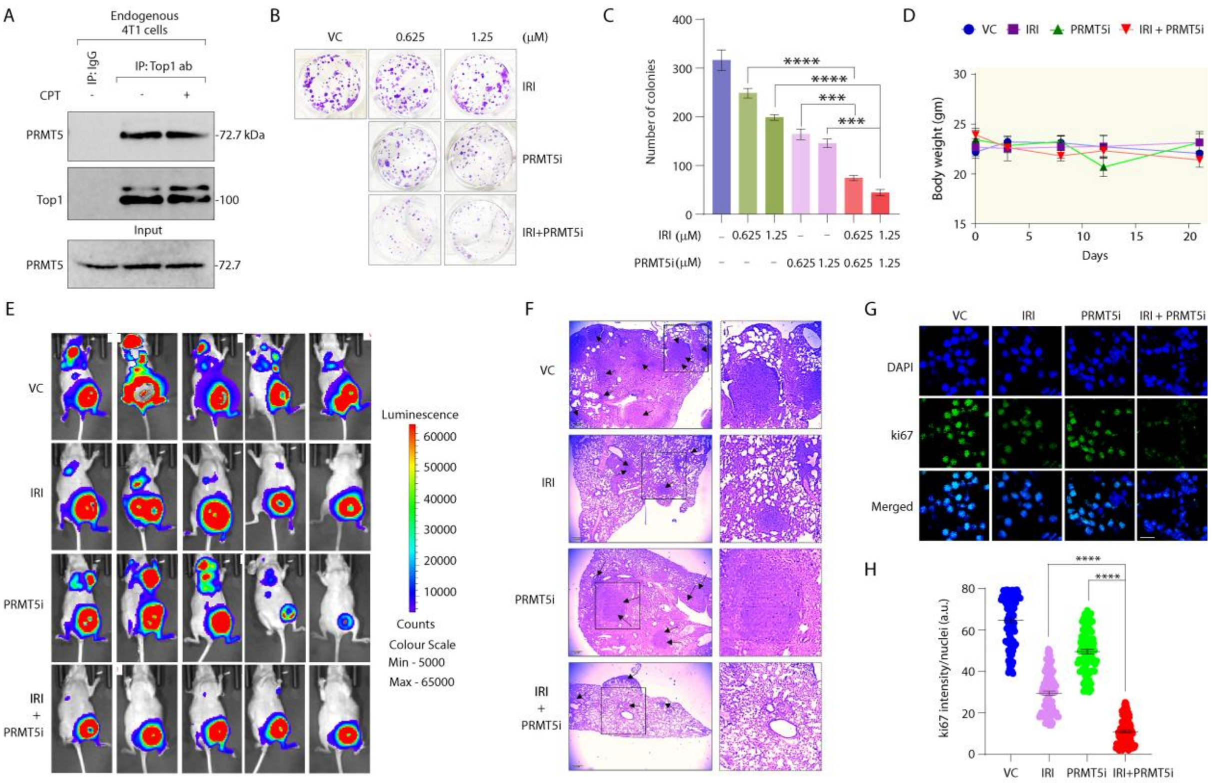

Figure S8: PRMT5i in combination with Top1 inhibitor inhibits tumor proliferation (A) Endogenous Top1 from Luc tagged 4T-1 cells treated with or without CPT (5 µM, 3 h) was immunoprecipitated using an anti-Top1 antibody, and the immunocomplexes were analyzed by immunoblotting with an anti-PRMT5 antibody. The blot was subsequently stripped and reprobed with anti-Top1 antibody to confirm uniform loading. Aliquots (10%) of the input lysates were immunoblotted to assess PRMT5 levels prior to immunoprecipitation. **(B)** Representative images showing crystal violet colony formation in Td-Tomato, Luc tagged 4T-1 cells treated with IRI or PRMT5i or both as indicated. **(C)** Graphical representation showing the number of colonies. Error bars represent mean ± SEM (n=3), ***P ≤0.001, ****P≤0.0001 (one-way ANOVA). **(D)** The body weight curve of mice treated with VC, IRI, PRMT5i, or IRI+PRMT5i. Data are shown as mean ± SEM, n=5. **(E)** *In vivo* bioluminescence monitoring of primary tumor and distant metastatic sites 21 days after treatment commenced. The color scale indicated bioluminescence intensity (counts/sec) emitted from each group (n=5) **(F)** Representative images of H&E-stained lung sections from mice treated with IRI, PRMT5i, or the combination of both, as indicated (left panel). Zoomed-in images of each group highlighting

Arginine Methylation of Human DNA Topoisomerase I by PRMT5 Facilitates DNA Relaxation

Saini Basu[1], Arpan Bhattacharyya[1,#], Muqtada Ali Khan[2,#], Uttam Pal[3], Srijita Paul Chowdhuri[1], Saumya Ranjan Satrusal[2, §], Laura Baranello[4], Dipak Datta[2, §] and Benu Brata Das[1*]

metastatic foci in the lungs are shown in the right panel. Scale bar, 500µm. **(G)** Immunohistochemistry was carried out on treated tumor samples to detect the expression of the proliferation marker Ki-67. Representative confocal images showing Ki-67 (green) and nuclei stained with DAPI (blue). Scale bar, 8µm. **(H)** Ki67 intensity was obtained from immunofluorescence confocal microscopy. Data are mean ± SEM, ****P≤0.0001 (t-test). Asterisks denote statistically significant differences. a.u. arbitrary unit.
